# Evaluation of the effect of olive extracts on blood pressure and cardiovascular health markers in adults: Findings from a double-blind, placebo-controlled, randomised trial

**DOI:** 10.1371/journal.pone.0344278

**Published:** 2026-03-10

**Authors:** Stef Lauwers, Annelies Breynaert, Annelies Verlaet, Erik Fransen, Tijs Bringmans, Lynn Roth, Emmy Tuenter, Johan Bosmans, Nina Hermans

**Affiliations:** 1 Natural Products & Food Research and Analysis - Pharmaceutical Technology (NatuRAPT), Department of Pharmaceutical Sciences, University of Antwerp, Wilrijk, Belgium; 2 Center for Medical Genetics, Faculty of Pharmaceutical, Biomedical, and Veterinary Sciences, University of Antwerp, Edegem, Belgium; 3 Department of Cardiology, Antwerp University Hospital, Edegem, Belgium; 4 Laboratory of Physiopharmacology, Department of Pharmaceutical Sciences, University of Antwerp, Wilrijk, Belgium; Kerman University of Medical Sciences, IRAN, ISLAMIC REPUBLIC OF

## Abstract

Despite advances in reducing cardiovascular disease (CVD) incidence, CVD mortality has increased, emphasising the need for new preventive strategies. Polyphenol-rich olive extracts have been proposed to lower blood pressure by reducing oxidative stress and enhancing nitric oxide production. This double-blind, placebo-controlled, randomised study aims to examine whether a standardised olive extract can lead to a reduction in blood pressure, lipid levels, and biological markers of oxidative stress after supplementation in the context of primary CVD prevention. In this trial, 56 participants with a systolic blood pressure ≥ 130 mmHg received capsules containing 440 mg olive dry extract (123.5 ± 9.4 mg oleuropein, 25.0 ± 3.8 mg hydroxytyrosol) or placebo on a daily basis over eight weeks. Both groups showed significant reductions in systolic blood pressure with a larger decrease in the intervention group as compared to the control group (intervention: −8.3 ± 2.2 mmHg, control: −7.3 ± 2.2 mmHg). However, this decrease was not found significantly different between the two groups. Moreover, total cholesterol, LDL, and apo B levels decreased in both groups, but with no significant difference between them. These results indicate that the olive extract was well-tolerated, although no significant hypotensive benefit was observed, with effects paralleling typical placebo responses in hypertension trials. Literature reports conflicting results on the hypotensive effect of olive polyphenols, indicating the need for further research with well-designed clinical trials. This trial was registered on Clinicaltrials.gov in April 2021, ID NCT04874961.

## 1. Introduction

Over the past few decades, remarkable progress has been made in developing strategies to lower the incidence of cardiovascular diseases (CVD). Despite these efforts, CVD remains the main cause of death and disability worldwide [1]. The declining CVD mortality rate that was observed remained stable in the last years or even increased in some populations [2,3]. Moreover, the actual burden of CVD has grown in the last twenty years by over 30%. This observed rise can largely be attributed to an increasingly ageing population [4]. With these concerning trends, the need for new actions to improve cardiovascular health remains critical.

Poor management of factors, such as diet, physical activity, blood lipids, and blood pressure, will increase the risk of developing CVD as a 20 mmHg increase in systolic blood pressure (SBP) is associated with a doubled risk of death from CVD [5,6]. CVD risk factors often occur clustered, as hypertension is linked to dyslipidaemia, impaired glucose tolerance and type 2 diabetes [7,8].

Implementing a healthy lifestyle can postpone or avert the onset of hypertension, reduce BP levels and lower the associated CVD risk [9]. The most effective lifestyle interventions are weight reduction [10], regular physical activity [11], smoking cessation [12] and dietary interventions [13]. In addition to a reduction of sodium intake [14], an increase in potassium intake [15], and a limited consumption of alcohol [16], certain dietary plans have been investigated. Diets, such as the Dietary Approaches to Stop Hypertension (DASH) Diet [17] and the Mediterranean Diet [18,19], are rich in whole grains, fruits, vegetables, and low-fat dairy products. The traditional Mediterranean diet also includes plenty of legumes, moderate portions of fish, low consumption of animal and *trans* fatty acids, and extra virgin olive oil as the main source of fat [17,19]. Previous research, including the PREDIMED trial, has shown that the Mediterranean diet lowers cardiovascular risk factors and, therefore, decreases the prevalence of CVD [20]. Due to the abundance of plant-based foods, the Mediterranean diet is rich in polyphenolic compounds, secondary metabolites to which health benefits, such as anti-oxidative and anti-inflammatory effects are attributed [21,22].

As mentioned, olives and derived products are prominently present in the Mediterranean diet and are a rich source of polyphenols. Compounds, such as secoiridoids (oleuropein, verbascoside), flavonoids (luteolin-7-*O*-glucoside, apigenin-7-*O*-glucoside, rutin), and simple phenols, like hydroxytyrosol and vanillic acid, have been detected in different parts of the olive tree (*Olea europaea* L., Oleaceae), including fruits and leaves. This diversity in polyphenols leads to a high antioxidant power of olive extracts [23,24]. Since oxidative stress plays a key role in the clinical manifestations of CVD [25], the anti-oxidative properties of polyphenols can be useful in the prevention of these conditions. In 2011, the European Commission approved a health claim stating that the consumption of olive oil polyphenols contributes to the protection of blood lipids from oxidative damage if at least 5 mg of hydroxytyrosol and its derivatives are consumed daily, which can be a beneficial physiological effect in preventing the initial development of CVD (primary prevention) as well as in reducing recurrence or complications in those with existing CVD (secondary prevention) [26].

In addition, in view of its cardioprotective activity, hypotensive properties have recently been reported for olive fruit and leaf extracts and olive oil rich in polyphenols [27–30], but additional research is necessary to strengthen this claim. The current study aims to examine whether a standardised olive extract can lead to a reduction in blood pressure, lipid levels, and biological markers of oxidative stress after daily supplementation over a period of eight weeks in the context of primary CVD prevention.

## 2. Methods

### 2.1 Trial design

A parallel randomised double-blind placebo-controlled clinical trial was conducted to investigate the short-term effect of a commercially available standardised olive extract in individuals with elevated blood pressure in relation to primary CVD prevention. This eight-week-long trial consisted of three visits. During a screening visit, the inclusion criteria were checked and factors that could have a potential effect on lipid levels and/or oxidative stress, such as smoking habits, physical exercise, menopausal status, and dietary habits, were recorded by a questionnaire. Outcome parameters were checked at the screening visit and after eight weeks of treatment. During a follow-up visit after four weeks of treatment, the blood pressure was measured.

#### 2.1.1 Randomisation.

Eligible participants were randomised into two groups, stratified by sex, and given a treatment of olive extract or placebo for eight weeks. The randomisation sequence was generated by the “Clinical Trial Randomization Tool” [31] with an arm allocation ratio of 1:1 and a maximum-tolerated imbalance of two.

A trained individual, who did not take part in the patient inclusion process and sample analysis, was responsible for the randomisation sequence and the coding of treatment packages. All other involved researchers and participants were masked from these actions to ensure the double-blind character of this trial.

#### 2.1.2 Sample size.

The expected effect size from the current study was based upon the results of a previous study investigating the effect of olive extract on systolic blood pressure found a decrease of 10.4 ± 11.4 mmHg without a significant change in the control group (−0.3 ± 6.3 mmHg) [29]. These data were relevant since a decrease in systolic blood pressure was the primary endpoint. The sample size was calculated using the previously reported effect size with a statistical power of 80%, and a dropout percentage of 10%. A significance level of 0.025 was used since a part of the participants of the placebo group originated from a different trial that investigates a different primary endpoint. This resulted in a requirement of 28 participants per group.

Calculations were executed by G*Power 3.1 [32].

#### 2.1.3 Ethical approval.

The clinical trial was performed in accordance with the ethical standards laid down in the 1964 Declaration of Helsinki and its later amendments. Ethical approval for the involvement of human subjects in this study was granted by the ethical review board of the Antwerp University Hospital on May 10^th^ 2021 and registered with the Belgian authorities (BUN 3002021000095) and clinicaltrials.gov in April 2021 (NCT04874961).

All participants agreed with and signed the written informed consent.

### 2.2 Participants

Participants were eligible if they had a systolic pressure of at least 130 mmHg, age between 18 and 77 years, and were able to ingest capsules. Exclusion criteria were: age < 18 or > 78 years, smoking, fasting triglyceride level > 400 mg/dL, weekly alcohol consumption > 14 units, with one unit corresponding to about 10 g of pure ethanol, chronic diseases, such as diabetes mellitus and rheumatoid arthritis, acute inflammation, desire to become pregnant or breastfeed during the trial, and the use of food supplements or medication that could influence the investigated outcome parameters, such as decongestants, triptans, and non-steroidal anti-inflammatory drugs.

Participants were still eligible if they discontinued the food supplements at least ten days before the screening visit. Participants who were chronically taking medication were allowed to partake in the trial if the medication and the condition did not interfere with the primary outcome of the trial and if the treatment regimen was already in place for a long time and was not going to change during the trial. As olive extracts could have potential as an add-on therapy in blood pressure reduction, participants using antihypertensive medication were included when the above-stated condition regarding the treatment regimen and the inclusion criteria were met. The participants were asked not to make any lifestyle changes, such as dietary patterns and level of physical activity, during the trial. Participants’ recruitment took place in Belgium between June 10^th^ 2021 and March 15^th^ 2024 through referral by general practitioners and flyer and email promotions. All participants provided written informed consent before participation in the study.

### 2.3 Intervention

This study compared the effect of a commercially available standardised olive extract with a placebo on systolic blood pressure. Participants were randomly divided into two groups and asked to take three capsules of the assigned study product with dinner for eight weeks. The three capsules were taken together to facilitate adherence to the intervention and to minimise the likelihood of missed doses.

The used olive dry extract is commercially available in Belgium as Tensiofytol® manufactured by Tilman (Baillonville, Belgium). A daily dose of the supplement consists of 334 mg olive leaf dry extract (standardised on 100 mg oleuropein), 106 mg olive fruit dry extract (standardised on 20 mg hydroxytyrosol), and auxiliary agents (microcrystalline cellulose, calcium phosphate, silicon dioxide, talc and magnesium salts of fatty acids). For this trial, the recommended daily amount was distributed over three opaque capsules.

The placebo consisted of microcrystalline cellulose, calcium phosphate, silicon dioxide, talc and magnesium salts of fatty acids. The same opaque capsules were filled with this powder to prepare the placebo.

Both treatments were prepared by the manufacturer.

As quality control, the concentration of oleuropein (OLE) and hydroxytyrosol (HT) was determined in the study product with an in-house validated high-performance liquid chromatography – electrochemical detection (HPLC-ECD) method adapted from Bayram et al. [33]. The description of this method can be found in the supplementary material, section S2.1 S2 File.

### 2.4 Outcomes

The primary outcome of this trial was to investigate the impact on systolic blood pressure after eight weeks of treatment.

The change from baseline in systolic and diastolic blood pressure after four weeks of treatment, in diastolic blood pressure after eight weeks of treatment, in oxidative stress markers (oxidised LDL (OxLDL), malondialdehyde (MDA), and glutathione (GSH)), in lipid profile (serum cholesterol, high-density lipoprotein (HDL), low-density lipoprotein (LDL), non-HDL, remnant cholesterol, triglycerides, lipoprotein(a) (Lp(a)), apolipoprotein A1 (apo A1), and apolipoprotein B (apo B)), and side effects after eight weeks were defined as secondary outcomes.

Exploratory outcomes were changes from baseline in glucose metabolism parameters (fasted glucose, haemoglobin A1c (HbA1c), insulin, and C-peptide) and a parameter correlated with cardiovascular risk (homocysteine) after eight weeks of treatment. Additionally, changes from baseline in ultra-sensitive C-reactive protein (CRP-US), creatinine, haemoglobin, estimated glomerular filtration rate (eGFR), waist circumference, and Body Mass Index (BMI) after eight weeks of treatment were investigated.

Blood pressure was measured by the researcher with a ProBP™ 2000 Digital Blood Pressure Device from Welch Allyn (Skaneateles Falls, NY, USA). All measurements were executed by the same researcher and device for each participant according to recommendations made by the American Heart Association [34] with a majority of the measurements performed in-office. A small fraction of the BP measurements were performed ambulatory to accommodate participants. Participants were instructed to be seated for at least five minutes before the first measurement. A minimum of three readings were taken at intervals of at least one minute. If a difference of more than 5 mmHg was noted between the readings, an additional measurement was done. The average of all readings was used.

Blood was collected in a fasted state in serum, K_3_-EDTA, FC Mix, and homocysteine detection Vacuette® tubes from Greiner Bio-One (Kremsmünster, Austria) at the screening visit and after eight weeks of treatment. The blood sampling was performed by a trained nurse.

Total cholesterol, HDL, and triglycerides were measured in serum by means of colourimetry. Apo A1, apo B and Lp(a) in serum, homocysteine in plasma from the homocysteine detection tube and glucose in plasma from the FC Mix tube were determined through spectrophotometry. Insulin and C-peptide levels were determined in serum with an electrochemiluminescence assay (ECLIA). CRP-US was measured in serum using immunoturbidimetry and used to assess inflammatory status. Using high-performance liquid chromatography with visible light detection (HPLC-VIS), HbA1c was measured in plasma. Since haemoglobinopathies and nephropathies influence HbA1c levels, haemoglobin in plasma and creatinine in serum were measured using colourimetry to better interpret the HbA1c values [35,36].

These measurements were performed in a certified clinical laboratory (‘Algemeen Medisch Laboratorium’, AML, Antwerp, Belgium) facilitated by the ‘Clinical Trials Department, AML’. The eGFR was calculated using the CKD-EPI equation [37] and used to evaluate the tolerance of the study product in terms of kidney function. Using the Friedewalde formula [38], the LDL concentration was obtained. Non-HDL was calculated by subtracting the HDL measurement from the total cholesterol measurement. Remnant cholesterol was determined by subtracting both the HDL value and the calculated LDL concentration from the measured total cholesterol.

Weight, height and waist circumference were measured during the screening visit by the researcher. Body weight was recorded using an analogue scale, while height and waist circumference were measured with a measuring tape. The BMI was calculated using the weight and height measurements.

For oxidative stress biomarker analysis, blood was collected in K_3_-EDTA tubes and immediately put on ice. Samples were centrifuged for 12 min at 2000 x g and 4°C. Plasma and red blood cells (RBC) were separated, aliquoted and kept at −80°C until analysis. The buffy coat layer was discarded. Both oxLDL and MDA were measured in duplicate in plasma according to the manufacturer’s instructions by an enzyme immunoassay kit, the Mercodia OxLDL sandwich ELISA kit (Mercodia AB, Uppsala, Sweden) and the MDA ELISA Kit (Elabscience, Wuhan, China). GSH was measured in duplicate in RBC according to an in-house validated method [39].

At the end of the study period, participants were asked to complete a questionnaire on lifestyle changes, compliance and side effects. Apart from self-reported side effects, muscle aches, muscle weakness, muscle stiffness, muscle cramps, arthralgia, insomnia, depression, hair loss, headache and obstipation were specifically questioned. The participants could indicate the severity of the side-effect as light, moderate, severe or very severe.

For the eight-week study period, 168 capsules were required. To ensure an adequate supply, participants received 210 capsules, sufficient for ten weeks of treatment. After completion of the study period, the remaining capsules were returned and counted by the researcher. The actual number of capsules taken was calculated by subtracting the number of returned capsules from 210. The percentage of compliance was calculated by dividing the number of capsules taken by the expected number of capsules taken.

### 2.5 Assessment of dietary intake

The average food intake of the participants was assessed by a validated Food Frequency Questionnaire (FFQ) [40] completed by the participant at the screening and end visit. Participants were asked to rate their average consumption frequency in the last three months of 50 food groups with a nine-point rating scale: never (0), less than one day a month (1), one to three days a month (2), one day a week (3), two to four days a week (4), five to six days a week (5), once a day (6), two to three times a day (7), or more than three times a day (8). Differences in dietary patterns at baseline between the two groups, shifts in dietary patterns within groups during the study period and the difference in shifts between the groups were assessed. Particular attention was given to foods rich in polyphenols, saturated and trans fatty acids, sugar, and sodium. An overview of the questioned food items and the composition and score calculation of the mentioned food groups can be found in the supplementary material, section S2.2 S2 File.

### 2.6 Statistical analysis

The comparison of baseline characteristics was performed in IBM SPSS Statistics for Windows, version 29.0.1.0 (IBM Corp., Armonk, NY, USA). Normality was checked with a Shapiro-Wilk test. Normally distributed outcomes were compared with an independent samples t-test and equality of variances was checked with Levene’s Test for Equality of Variances. The Fisher’s Exact Test was used to compare categorical variables between the two groups. For the baseline comparison of not normally distributed parameters and the FFQ, a Mann-Whitney U test was used.

The change from baseline after eight or four weeks of treatment was investigated using a linear mixed model with time point, treatment group, and the interaction of time point and treatment group as fixed effects and participant as random effect. Homoscedasticity and normal distribution of the residuals were checked visually. Subgroup analysis of the primary outcome parameter by sex or the use of blood pressure medication was performed with a linear mixed model for each treatment group using sex or blood pressure medication, time point, and the interaction term sex*time point or blood pressure medication*time point as fixed effects and participant as a random effect.

To investigate the influence of the starting value of the primary outcome on the observed effect, least squares regression was used with the end value as the outcome parameter and starting value, treatment group and the interaction between the starting value and treatment group as independent variables.

These analyses were performed in JMP Pro for Windows, version 17.2.0 (SAS Institute Inc., Cary, NC, USA). Data of participants who dropped out of the trial or with a calculated compliance < 80% were excluded from data analysis.

Data analysis of the GSH quantification was performed using Microsoft Excel (Microsoft, Redmond, WA, USA) and GraphPad Prism for Windows, version 9.4.1 (GraphPad Software, Boston, MA, USA) for OxLDL and MDA quantification according to the manufacturer’s instructions.

To assess a potential shift in dietary intake between the start and end of the study period within a group, a Wilcoxon signed rank test was performed on the FFQ scores from the screening and end visit for each questioned food item and food group. To compare the difference in dietary intake shift during the study between the two groups, a Mann-Whitney U test was performed on the difference in scores of the screening and end visit. Comparisons were performed in IBM SPSS Statistics.

## 3. Results

### 3.1 Participant recruitment and baseline comparison

From June 2021 to March 2024, 56 participants who met the inclusion criteria were randomly allocated to the intervention group (n = 28) or the control group (n = 28). Even though all participants completed the trial, one participant in each group had a compliance percentage of less than 80% and was thus excluded from the statistical analysis of the outcomes.

**Fig 1:**
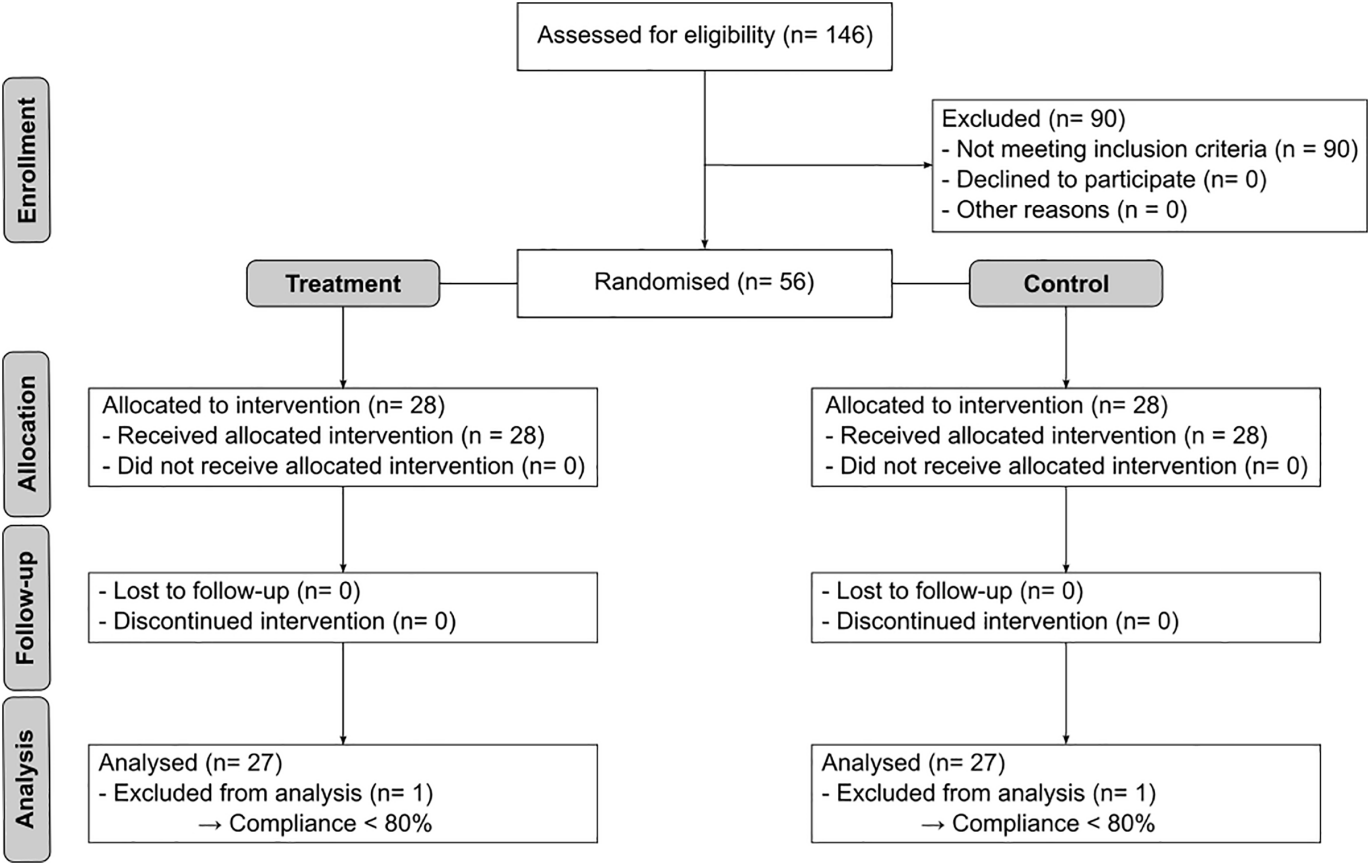
Flow diagram of participants in the study.

The average compliance (expressed as mean ± standard error) in the intervention group was 96.1 ± 0.9% and 95.4 ± 1.1% in the control group and did not differ significantly between the two groups (p = 0.762). Fig 1 depicts the patient flow during this trial. No statistically significant differences were found between the intervention and placebo groups at baseline (Table 1).

**Table 1:** Baseline characteristics of study participants. Values of continuous parameters are depicted as the mean with the corresponding standard deviation between brackets.

Characteristics	Intervention (n = 28)	Control (n = 28)	p-value
Age	54.3 (13.4)	58.5 (11.0)	0.208
Sex	Male: 14	Male: 17	0.591
	Female: 14	Female: 11	
Hypertension medication	11/28	13/28	0.787
Cholesterol medication	2/28	3/28	1.000
Other medication	10/28	9/28	1.000
Food supplements	5/28	3/28	0.705
Postmenopausal status in women	12/14	8/11	0.623
Systolic blood pressure (mmHg)	147.6 (12.7)	148.0 (14.9)	0.850
Diastolic blood pressure (mmHg)	92.2 (7.8)	80.5 (8.5)	0.255
Weight (kg)	81.0 (16.8)	87.7 (17.2)	0.144
BMI (kg/m²)	28.0 (4.5)	28.7 (5.1)	0.565
Waist circumference (cm)	101.0 (13.0)	103.6 (12.4)	0.439
Total cholesterol (mg/dL)	254.6 (116.6)	236.3 (32.6)	0.825
HDL (mg/dL)	53.3 (12.6)	59.2 (18.6)	0.174
LDL (mg/dL)	149.2 (45.7)	152.5 (30.9)	0.757
Non-HDL (mg/dL)	179.8 (53.5)	177.1 (33.6)	0.821
Remnant cholesterol (mg/dL)	30.5 (17.3)	24.6 (10.5)	0.426
Triglycerides (mg/dL)	157.9 (85.4)	122.5 (52.3)	0.269
Apo A1 (g/L)	1.6 (0.3)	1.6 (0.3)	0.577
Apo B (g/L)	1.19 (0.07)	1.19 (0.04)	0.954
Lp(a) (mg/L)	137.1 (203.4)	170.2 (237.2)	0.849
OxLDL (U/L)	78.0 (3.9)	74.7 (9.9)	0.551
MDA (ng/mL)	506.8 (79.0)	490.7 (79.8)	0.788
GSH (µg/mL RBC)	549.6 (22.6)	607.4 (33.0)	0.196
Glucose (mg/dL)	101.1 (34.8)	94.6 (14.5)	0.258
HbA1c (%)	5.8 (0.2)	5.56 (0.07)	0.748
Homocysteine (µmol/L)	8.0 (4.0)	8.6 (8.3)	0.351

Eleven patients in the intervention group and thirteen patients in the control group used antihypertensive medication such as ACE inhibitors, Angiotensin II receptor blockers, beta-blockers, diuretics and calcium channel blockers. Three patients in the control group and two in the intervention group used lipid-lowering medication; all of them were statins. Other reported medications were proton pump inhibitors, oestrogen-progestogen combinations (oral contraceptives and hormone replacement therapy), inhaled corticosteroids in combination with long-acting β2 agonists, and antihistamines (chronic use). Additionally, the use of vitamin B12 and D, and zinc and magnesium supplements was documented.

Analysis of the study product showed that 3 capsules contained 123.5 ± 9.4 mg OLE and 25.0 ± 3.8 mg HT, which corresponds respectively with 123.5 ± 9.4% and 125.2 ± 18.9% of the concentration mentioned by the manufacturer.

### 3.2 Outcomes

**Fig 2:**
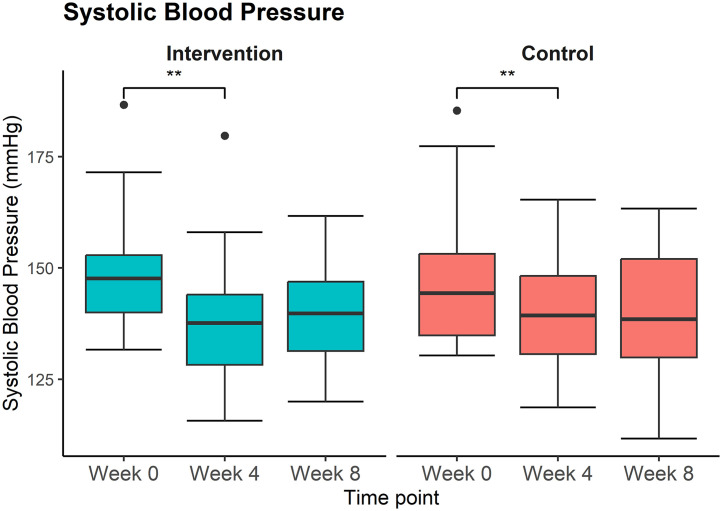
Boxplots of systolic blood pressure for each time point of control (red) and intervention (green) groups. Significance is indicated with brackets and two asterisks (**) when the p-value is < 0.01.

Tables 2 and 3 display the mean value and standard error of the outcome parameters at baseline and after eight weeks of treatment. Table 2A also shows systolic and diastolic blood pressure results after four weeks of treatment. Tables 2B and 3 show the mean difference of the outcome parameters and the *p*-value of the interaction term from the mixed model. The systolic blood pressure, as the primary outcome, decreased by 8.3 ± 2.2 mmHg in the intervention group and by 7.3 ± 2.2 mmHg in the control group. Fig 2 is a visual depiction of the systolic blood pressure values at each time point. Although the value after eight weeks of treatment is significantly lower than the baseline value (p < 0.0001), the observed effect is not statistically different between the two groups. Further analysis showed that the decrease in blood pressure occurred within the first four weeks of treatment. Subgroup analysis revealed that sex (treatment p = 0.794; control p = 0.613), the use of blood pressure medication (treatment p = 0.504; control p = 0.931), and baseline value (p = 0.320) had no significant influence on the effect. The same observations were made for diastolic blood pressure.

**Table 2:** Mean values with standard error of systolic and diastolic blood pressure at baseline, after 4 weeks, and after 8 weeks of treatment for the intervention and control group (A) together with the mean values with standard error and percentage of the raw difference in study outcomes for the intervention and control group with the corresponding p-value of the mixed model interaction term (B).

A	Intervention (n = 27)			Control (n = 27)		
	Baseline	4 weeks	8 weeks	Baseline	4 weeks	8 weeks
Systolic blood pressure (mmHg)	148.0 (2.5)	137.4 (2.7)	139.8 (2.0)	147.3 (2.8)	139.6 (2.3)	140.0 (2.6)
Diastolic blood pressure (mmHg)	92.2 (1.5)	86.1 (1.9)	87.5 (1.6)	89.9 (1.6)	85.3 (1.9)	86.6 (1.7)
**B**	**Intervention** **(n = 27)**	**Control** **(n = 27)**				
	**Raw difference (SE)**	**Raw difference (SE)**	***p*-value**			
**Primary outcome**						
Difference in systolic blood pressure after 8 weeks (mmHg)	−8.3 (2.2)	−7.3 (2.2)	0.760			
**Secondary outcomes**						
Difference in systolic blood pressure after 4 weeks (mmHg)	−10.6 (2.5)	−7.8 (2.4)	0.780			
Difference in systolic blood pressure between 4 and 8 weeks (mmHg)	2.4 (2.3)	0.5 (2.8)	0.581			
Difference in diastolic blood pressure after 8 weeks (mmHg)	−4.7 (1.0)	−3.3 (0.9)	0.302			

**Table 3:** Mean values with standard error of secondary and exploratory outcomes at baseline and after 8 weeks of treatment for the intervention and control group, together with the mean values with standard error and percentage of the raw difference in study outcomes for the intervention and control group, with the corresponding p-value of the mixed model interaction term. P-values < 0.05 are indicated with an asterisk (*).

	Intervention (n = 27)		Control (n = 27)		Difference Intervention		Difference Control		p-value
	Baseline	8 weeks	Baseline	8 weeks	Raw difference (SE)	%	Raw difference (SE)	%	
**Secondary outcomes**									
Total cholesterol (mg/dL)	236.6 (9.2)	222.8 (7.9)	237.8 (6.2)	230.2 (8.4)	−13.8 (3.7)	−5.8%	−7.7 (5.6)	−3.2%	0.372
HDL (mg/dL)	54.1 (2.4)	53.5 (2.6)	59.6 (3.6)	57.4 (3.6)	−0.6 (1.1)	−1.0%	−2.2 (1.3)	−3.7%	0.358
LDL (mg/dL)	151.3 (8.7)	136.7 (7.1)	153.5 (6.0)	144.7 (7.6)	−11.2 (4.1)	−7.4%	−9.0 (4.3)	−5.9%	0.645
Non-HDL (mg/dL)	182.5 (10.1)	169.3 (9.0)	178.3 (6.5)	172.7 (8.7)	−11.8 (3.1)	−6.5%	−8.4 (4.7)	−4.7%	0.226
Remnant cholesterol (mg/dL)	31.2 (3.3)	29.6 (3.0)	24.8 (2.0)	25.8 (2.9)	−0.6 (2.2)	−2.1%	1.7 (2.9)	6.7%	0.504
Triglycerides (mg/dL)	161.2 (16.4)	157.7 (17.4)	123.6 (10.)	140.0 (20.2)	−3.4 (9.5)	−2.1%	16.4 (16.4)	13.3%	0.300
Apo A1 (g/L)	1.61 (0.05)	1.59 (0.06)	1.64 (0.06)	1.64 (0.06)	−0.03 (0.03)	−1.86%	−0.01 (0.03)	−0.6%	0.547
Apo B (g/L)	1.22 (0.04)	1.12 (0.05)	1.22 (0.04)	1.19 (0.05)	−0.11(0.03)	−9.0%	−0.03 (0.03)	−2.5%	0.043*
Lp(a) mg/L	141.4 (39.7)	137.5 (40.0)	175.7 (46.2)	168.5 (50.8)	−3.9 (6.4)	−2.7%	−7.2 (20.2)	−4.1%	*0.875*
OxLDL (U/L)	75.4 (4.1)	72.4 (4.2)	77.6 (3.7)	76.9 (6.0)	−3.0 (2.0)	−4.0%	−0.7 (3.5)	−0.8%	0.345
MDA (ng/mL)	591.4 (94.3)	529.0 (72.0)	406.6 (54.6)	462.7 (65.6)	−62.4 (42.9)	−10.6%	56.0 (23.4)	13.8%	0.603
GSH (µg/mL RBC)	585.9 (32.2)	583.0 (19.9)	566.8 (23.2)	589.4 (14.4)	−2.9 (20.1)	−0.5%	22.7 (18.3)	4.0%	0.049*
**Exploratory outcomes**									
Glucose (mg/dL)	101.7 (6.8)	99.2 (6.1)	94.8 (2.8)	96.7 (2.5)	−2.4 (1.4)	−2.4%	2.0 (1.5)	2.1%	0.040*
HbA1c (%)	5.8 (0.2)	5.8 (0.2)	5.58 (0.07)	5.62 (0.06)	−0.05 (0.03)	−0.9%	0.01 (0.03)	0.2%	0.132
Homocysteine (µmol/L)	8.2 (0.8)	7.9 (0.8)	8.8 (1.6)	6.4 (0.5)	−0.3 (0.4)	−3.5%	−2.5 (1.3)	−28.7%	0.119

Results of the secondary outcomes, markers of oxidative stress and lipid profile are displayed in Table 3. A slight increase in GSH levels in the control group was noticed, while the levels in the treatment group remained unchanged. However, this difference was only nominally significant (p = 0.049). No significant changes in MDA and oxLDL levels were observed. Cholesterol values decreased in both intervention and control groups. Even though this change is not significantly different between the groups, the measured values of total cholesterol, LDL and non-HDL at the end of the study were significantly lower than at the start with p-values of 0.0024, 0.001, and 0.0045 for total cholesterol, LDL, and non-HDL, respectively. A larger decrease in apo B values was seen in the treatment group as compared to the control group, leading to a nominally significant p-value of 0.043. Elimination of the interaction term in the mixed model analysis revealed that apo B is significantly lower after eight weeks in both groups (p = 0.0014). The levels of remnant cholesterol, HDL, triglycerides, apo A1, and Lp(a) remained unchanged.

The exploratory parameter glucose showed a nominally significant difference (p = 0.040) between the two groups, with a decrease in the intervention group and an increase in the control group. However, HbA1c levels did not differ between time points and between the groups. Additionally, homocysteine values were unaffected. The results of the other prespecified exploratory parameter can be found in section S2.3 of the supplementary material S2 File.

### 3.3 Assessment of dietary intake

At baseline, no difference in intake of certain food groups was detected. When investigating the food intake during the trial period, a statistically significant increase in diet soft drinks from 1.9 ± 1.8 to 2.4 ± 2.3 (p = 0.030) and a decrease in consumption of boiled potatoes from 3.6 ± 0.9 to 3.2 ± 1.2 (p = 0.017) were found in the control group. In the intervention group, a significant decrease in the consumption of yoghurt from 4.4 ± 1.9 to 3.8 ± 1.6 (p = 0.012) and a decrease in the consumption of game meat from 0.7 ± 0.7 to 0.4 ± 0.6 (p = 0.011) were observed. However, the changes in dietary intake were not statistically different between the two groups.

When investigating the intake of foods rich in polyphenols, saturated and/or trans fatty acids, sugar, and sodium, no differences in intake at baseline between the study groups and during the trial period within or between the study groups were found.

### 3.4 Side effects

No significant differences were observed in the prevalence of side effects between the two groups.

16/27 (59.26%) of participants in the treatment group and 21/27 (77.78%) of participants in the control group did not report any effects. eGFR values did not differ between groups at the start and the end of the study and did not change during the trial period.

The most commonly reported side effects were muscle pain (7.41%), headache (7.41%), and sleeplessness (5.55%).

All reported side effects were resolved by the end of the trial or did not require any treatment or follow-up.

A more detailed overview of the reported side effects can be found in section S2.4 of the supplementary material S2 File.

## 4. Discussion

In this double-blind, placebo-controlled, randomised clinical trial, the effect of eight weeks of supplementation with a commercially available olive extract was investigated on BP, lipid profile, and biomarkers of oxidative stress and CVD in participants with an SBP ≥ 130 mmHg in the context of primary CVD prevention.

The pathophysiology of hypertension is complex and is a result of an intricate interplay between genetic and environmental factors, such as lifestyle, air pollution, immuno-inflammation, oxidative stress, nitric oxide (NO) production, alterations in the renin-angiotensin-aldosterone system (RAAS), endothelial dysfunction, and gut microbiome [41].

Olive polyphenols could interact with several of these elements. The most apparent mechanism of action is the potential of olive polyphenols to reduce oxidative stress by direct scavenging of oxidants, thereby reducing the level of reactive oxygen and nitrogen species, which contribute to hypertension [42]. However, it is more likely that the interaction with membrane receptors and/or enzymes involved in cell signalling results in the indirect alteration of the cellular redox status [43]. It is demonstrated that olive polyphenols decrease nicotinamide adenine dinucleotide phosphate (NADPH) oxidase activity and therefore, reduce oxidative stress [44,45]. In addition, studies showed that olive polyphenols can increase endothelial nitric oxide synthase (eNOS) production [46], decrease the expression of inducible NOS (iNOS) in pathophysiological conditions [47,48]. Literature also shows that olive polyphenols decrease the expression of proinflammatory cytokines, like interleukin 1β (IL-1β) [49].

Even though research proposes different possible mechanisms of action, in this trial, no overall significant difference was found in SBP reduction between the intervention and control group. Although a significant decrease of −8.3 ± 2.2 mmHg in the intervention group and −7.3 ± 2.2 mmHg in the control group was observed between the start and end of the trial, the overall difference was not significant since the reduction occurred in both groups.

A long-term study, which lasted over four years, comparing the effect of the Mediterranean diet and a low-fat diet [19], and a four-month study comparing the Mediterranean diet with a vegan diet [50], reported a decrease in BP in individuals with an elevated CVD risk. Although BP decreased significantly in all diets, a greater reduction in BP was observed in individuals adhering to the Mediterranean diet as compared to a vegan diet [50], and diastolic BP was lower in individuals who consumed the Mediterranean diet compared to the low-fat diet [19]. Additionally, Davis et al. reported a small but significant decrease in systolic BP in healthy Australian individuals over 64 years of age who consumed a Mediterranean diet for six months as compared to their habitual diet [51]. A study comparing the supplementation of the Mediterranean diet with a polyphenol-rich or polyphenol-free olive oil for four months in young women with mild hypertension observed a significant decrease in BP in the polyphenol-rich olive oil group, while BP persisted in the polyphenol-free olive oil group [52]. A shorter, twelve-week study investigated the influence of a healthy US Mediterranean and vegetarian diet on body weight, HbA1c, and BP in African American individuals at risk of type 2 diabetes mellitus, and only found a significant decrease in BP in the healthy US diet intervention group [53]. However, a meta-analysis on the influence of the Mediterranean diet enriched with olive oil showed no consistent benefit on blood pressure [54].

When further focussing on olive oil and its phenolic constituents, a three-week cross-over trial showed that BP is reduced after consumption of high-polyphenolic olive oil as compared to low-polyphenolic olive oil [55] and that virgin olive oil reduces SBP in hypertensive elderly individuals in a four-week cross-over trial, while consuming sunflower oil has no effect [56]. In contrast, multiple meta-analyses investigating the effect of high-polyphenolic olive oil on blood pressure reported no significant [57,58] or only a small beneficial effect on SBP [59].

Clinical trials using olive extracts report conflicting results about their hypotensive properties. Studies that find a significant result report a 3–10 mmHg decrease in SBP upon receiving a daily amount of 80–136 mg of oleuropein and/or 6–10 mg of hydroxytyrosol, ingested at once or in two doses, throughout six to twelve weeks of treatment in the intervention group, while no difference was observed in the placebo group [29,60–63]. The studies conducted by Hermans et al. and Perrinjaquet-Moccetti et al., where participants received a daily amount of 100–200 mg of oleuropein and/or 20 mg of hydroxytyrosol, ingested at once or in two doses, found a decrease of 13 mmHg and 6 mmHg in SBP, respectively, but these trials were not placebo-controlled [27] or the control group received lifestyle advice instead of treatment [64]. Other studies with daily doses between 50 and 200 mg of oleuropein and/or 10 mg of hydroxytyrosol taken in one dose, report no effect in both intervention and placebo groups [65–67].

However, some remarks can be made when comparing the results of these studies. Firstly, it is important to mention that BP is not always the primary outcome parameter in these studies [29,63,65,66]. Often lipid profiles and/or multiple markers for CVD were investigated, leading to the inclusion of participants with normal to only slightly elevated BP levels, which makes the observation of a decrease in BP less likely. Furthermore, the sample size used may not be suitable for observing differences in BP, since its calculation is based on the primary objective of the trial.

Secondly, the accurate measurement of BP is of great importance since there is often a large variability between BP readings. The most common methods of BP measurement are the 24-hour ambulatory measurement and the in-office measurement using a manual or digital sphygmomanometer. During a 24-hour measurement, the BP is measured at thirty-minute to one-hour intervals, providing multiple readings in conditions reflecting the usual environment [41]. Although this is a reliable method to evaluate BP, it is less patient-friendly, less cost-effective, and more time-consuming than in-office measurements [68]. In-office BP measurements are subject to the ‘white-coat effect’ and are therefore known to be consistently higher than measurements at home. This phenomenon can be minimised by performing multiple standardised measurements over repeated visits [69], illustrating the importance of following official guidelines [34,41].

Although, understandably, adherence to measurement guidelines varies when BP is not a primary outcome parameter, the applied measuring protocol should be well described when reporting results, acknowledging the influence of the measuring method on BP values. In this study, measurement with a digital sphygmomanometer, predominantly in-office, was chosen as the preferred method because of its user-friendliness, cost-effectiveness, and convenience. The above-mentioned studies with BP as the primary outcome used 24-hour ambulatory measurements [60,61], measurements with a sphygmomanometer only during study visits [27,62,64], or a combination of both methods [67]. Often, it was not disclosed if the measurements during study visits were performed in-office or ambulatory.

Thirdly, the composition of the studied olive extract varies between the trials. Olive extracts, predominantly olive leaf extracts, are often standardised on their oleuropein content and in the mentioned studies, the daily oleuropein intake ranges from 50 to 200 mg or their dose was not provided. Some studies used a product where olive extract was combined with other plant extracts [29,63,67]. The variation in the content of oleuropein, as well as other polyphenols, can influence the observed outcome of these trials [70].

Since blood pressure is directly linked to cardiovascular health and its measuring methods are non-invasive, cost-effective and well-established in standardised guidelines, it is a highly relevant parameter. However, the strong heterogeneity in results complicates the formulation of a general conclusion regarding the hypotensive effects of olive polyphenols. To assess the BP-lowering properties of the constituents of *Olea europaea* L., larger-scale, well-designed, randomised controlled trials with BP as a primary outcome are essential.

In the current trial, a significant decrease in SBP from baseline was observed in both the intervention and control groups after an eight-week treatment period. However, this observation aligns with findings commonly reported in similar trials. Patel et al. investigated the magnitude of BP reduction in placebo arms of hypertension trials published up to June 2014, focusing on five antihypertensive drugs licensed by the US Food and Drug Administration since 2000 and found a decrease in SBP of ≈ 6 mmHg in trials of non-resistant hypertension and ≈ 9 mmHg in trials of resistant hypertension. If valid, this order of reduction is clinically relevant, since it would diminish the risk of stroke by 14% and give a 7% reduction in mortality [71]. Research confirms that the placebo effect in clinical trials investigating antihypertensive treatments is of importance. A meta-analysis of trials using beta-blockers revealed that placebo effects on SBP accounted for 34% of the drug effect [72]. The observed decrease in SBP of 8.3 ± 2.2 mmHg in the intervention group and 7.3 ± 2.2 mmHg in the control group in our eight-week trial can, therefore, not be ascribed to the olive extract. The study by Patel et al. also reported that age, trial duration, washout/run-on period duration, and dropout rate had no significant effect on the observed change in BP, indicating that prolonging the clinical trial would not reduce the magnitude of the placebo effect. In this clinical trial, the decrease in SBP happened rather quickly, within the first four weeks of treatment, strengthening the assumption that the observed change in BP is attributed to the placebo effect [71]. Since the BP did not change significantly between four and eight weeks of treatment, extending the trial period would only lead to a different result if the potential additional effect of the intervention became more visible after a longer treatment period. Measures can be taken to reduce or take into account the placebo effect, such as applying a single-blind period at the start of the trial during which all participants receive a placebo [73,74] or using ambulatory BP measurements, albeit in combination with in-office measurements, as this results in smaller BP reductions in placebo arms [67,71].

The effect size reported in the study by Verhoeven et al. on which the sample calculation is based [29], corresponds to a standardised effect size (Cohen’s d) of 0.91. Since the current study observed a standard deviation of 11.4 mmHg (SE*sqrt(n)) (Table 2B), a difference in means of 10.4 mmHg between the placebo and intervention group can be detected with 80% power. In this case, the observed non-significant result means that the difference between the two groups is smaller than 10.4 mmHg. Therefore, these results cannot exclude a more subtle effect of the treatment on the outcome.

The influence of olive polyphenols on oxidative stress biomarkers has been well described in the literature [49,75–77], leading to the endorsement of the aforementioned health claim by EFSA [26].

In the current clinical trial, lower MDA and oxLDL levels in the intervention group were seen; however, these differences were not significant, although previous studies report a reduction of these biomarkers of lipid oxidation [52,78–81].

The comparison of GSH levels during this trial between the two treatment groups resulted in a *p*-value of the interaction term of 0.049. However, since this nominally significant result will not survive any multiple testing correction and because GSH is a secondary parameter, no conclusion could be drawn from these findings.

Many studies on the effect of olive polyphenols on the blood lipid profile use olive oil in their study design, which makes it difficult to differentiate between the effect of unsaturated fats and the olive polyphenols on the blood lipid levels. A subanalysis of the aforementioned PREDIMED trial, investigating the effect of adherence to the Mediterranean diet or a low-fat diet after one year in 284 individuals with three or more cardiovascular risk factors, reported a significant decrease in total cholesterol of 11.3 mg/dL [–16.8; –5.7] in participants following the Mediterranean diet supplemented with extra virgin olive oil [18]. However, a meta-analysis on the effect of daily exposure to olive oil polyphenols on lipid markers concluded that there was no significant effect on total cholesterol, a slight reduction of –4.28 mg/dL [–5.78; –2.77] in LDL cholesterol when 320−600 mg/kg olive oil polyphenols were consumed daily and a significant increase of 1.13 mg/dL [0.45; 1.80] of HDL levels independent of the daily consumption level [82]. Additionally, a meta-analysis studying the effect of olive leaf extract on cardiovascular risk factors reports a significant reduction in total cholesterol of −9.14 mg/dL [−13.80;-4.47], in LDL cholesterol of −4.60 mg/dL [−8.26; −0.94], and in triglyceride levels of −14.32 mg/dL [−19.36; −9.28] in patients with hypertension [28]. If these changes are clinically relevant, remains uncertain. Research on the effect of olive polyphenols on apo A1, apo B, and Lp(a) levels is limited and reported no effect on these parameters [83,84].

In the current trail, outcome parameters linked to the lipid profile were not significantly different between both groups. Similarly, as mentioned for the GSH result, the corresponding p-value of apo B (p = 0.043) would not be considered significant. Nevertheless, the values for total cholesterol, LDL, non-HDL, and apo B decreased significantly during the trial. Since this decrease was observed in both groups, the detected effect cannot be ascribed to olive polyphenols. A lifestyle change could be responsible for this phenomenon, although no participants declared to have changed their diet or level of physical activity during the trial. The assessment of dietary intake revealed some statistically significant differences in intake between the start and end of the trial, like an increase in the consumption of soft drinks in the control group and a decrease in yoghurt consumption in the control group. However, none of these findings were clinically relevant. This discrepancy between the change in cholesterol levels and the results of the FFQ casts doubts on the accuracy with which the participants responded to the questionnaire. Dietary assessment is very important when performing research on supplements or evaluating parameters that may be influenced by diet. Established methods are food records, FFQs, and 24-hour recalls. FFQs are more cost-effective since participants can complete them autonomously [85]. A combination of a 24-hour dietary recall and an FFQ has been used to increase the accuracy [86]. People tend to underreport foods with a negative health image and over-report those with a positive connotation [87]. Ideally, the diet should be controlled during this type of trial or the use of validated biomarkers to assess the intake of certain nutrients could be a more feasible approach to help improve the accuracy of dietary intake questionnaires.

Since the relation between sodium intake and hypertension risk is well established [88], investigating its consumption could yield valuable information. Therefore, the scores of sodium-rich food items from the validated FFQ were combined to evaluate the potential differences in salt intake during the trial period. The statistical analysis did not reveal any differences between and within the study groups at baseline and during the study. However, this result requires some contextualisation. In addition to the aforementioned limitations of the FFQ, this questionnaire was not optimised to comprehensively evaluate sodium intake since it does not specifically mention prepared and ready-made meals, which are the largest contributors to total sodium consumption [89]. Also, no questions about discretionary salt use were included.

The comparison of the change in fasted glucose levels between the two groups resulted in a p-value of 0.040. Although this is an interesting observation, more research is necessary to investigate the effect of olive polyphenols on glucose levels. Additionally, the reported difference would not be considered significant after correction for multiple testing. Even though literature reports positive effects on glucose regulation after twelve weeks of supplementation with olive leaf polyphenols in middle-aged, overweight men [65], clinical evidence on the anti-diabetic effect of olive polyphenols is too preliminary, making clinical trials, designed specifically to investigate this activity, pivotal [90]. HbA1c levels, which reflect the average of blood glucose over the previous two to three months, remained unchanged during the trial. Considering the eight-week trial period and the negligible change in fasted glucose, this result was predictable. Additionally, it is also important to note that participants in this trial did not necessarily have elevated fasting glucose or HbA1c levels.

Homocysteine levels, associated with an increased risk of CVD when elevated, did not differ during the trial and between the two groups. The average baseline levels of homocysteine were not elevated. Other prespecified exploratory parameters are discussed in section S2.3 of the supplementary material S2 File.

The supplement was well tolerated and only a minority of participants reported some side effects during the trial. The declared side effects were of minor severity. However, the complaints mentioned by the intervention group could not be ascribed to the olive leaf extract. No significant differences between intervention and control groups were detected in the prevalence and severity of side effects. The comparison of the eGFR values between the two groups did not reveal any changes in this parameter, indicating that kidney function did not diminish during the trial period.

## 5. Conclusion

In this double-blind, placebo-controlled, randomised clinical trial, the effect of an eight-week supplementation with a commercially available olive extract on BP, lipid profile, and biomarkers of oxidative stress and CVD was investigated in participants with a SBP ≥ 130 mmHg. The supplement was well-tolerated with only minor reported side effects. A significant decrease in SBP was observed between the start and end of the trial in both the intervention (−8.3 ± 2.2 mmHg) and the control group (−7.3 ± 2.2 mmHg). The reported change was not significantly different between the two groups. The effect size is comparable with the SBP reduction in placebo arms in hypertension trials. Therefore, the hypotensive properties of olive polyphenols could not be confirmed in this trial. The combination of 24-hour ambulatory and in-office BP measurements and the application of a single-blind placebo period at the start of the trial could have reduced the placebo effect. The conflicting results in the literature illustrate the need for well-designed clinical trials that investigate the effect of olive polyphenols on BP, with BP as the primary outcome and standardised methods of BP measurement.

## Supporting information

S1 File CONSORT checklist. (PDF)

S2 File Supplementary material. (PDF)

S3 File Study protocol. (PDF)

## Abbreviations

Apo A1: Apolipoprotein A1
Apo B: Apolipoprotein B
BP: Blood pressure
CKD-EPI: Chronic Kidney Disease – Epidemiology Collaboration
CRP-US: C-reactive protein – ultra sensitive
CVD: Cardiovascular diseases
DASH: Dietary Approaches to Stop Hypertension
ECLIA: Electrochemiluminescence assay
EFSA: European Food Safety Authority
eGFR: Estimated glomerular filtration rate
ELISA: Enzyme-linked immunosorbent assay
eNOS: Endothelial nitric oxide synthase
FFQ: Food frequency questionnaire
GSH: Glutathione
HbA1c: Haemoglobin A1c
HDL: High-density lipoprotein
HPLC-ECD: High-performance liquid chromatography – electrochemical detection
HPLC-VIS: High-performance liquid chromatography – visible light detection
HT: Hydroxytyrosol
IL-1β: Interleukin 1β
iNOS: Inducible nitric oxide synthase
LDL: Low-density lipoprotein
Lp(a): Lipoprotein(a)
MDA: Malondialdehyde
NADPH: Nicotinamide adenine dinucleotide phosphate
OLE: Oleuropein
OxLDL: Oxidised low-density lipoprotein
RAAS: Renin-angiotensin-aldosterone system
RBC: Red blood cells
SBP: Systolic blood pressure
