# Supplementary material for: Evaluation of the effect of olive extracts on blood pressure and cardiovascular health markers in adults: Findings from a double-blind, placebo-controlled, randomised trial
Source: PLoS One. 2026 Mar 10;21(3):e0344278. doi: 10.1371/journal.pone.0344278 (PMC12974854; doi:10.1371/journal.pone.0344278)
Supplement: S2 File — (PDF) [file pone.0344278.s002.pdf]

Evaluation of the Effect of Olive Extracts on Blood Pressure and Cardiovascular Health Markers: Findings from a Double-blind, Placebo-controlled, Randomised Trial

Lauwers Stef[a], Breynaert Annelies[a], Verlaet Annelies[a], Fransen Erik[b], Bringmans Tijs[c], Lynn Roth[d] Tuenter Emmy[a], Bosmans Johan[c], Hermans Nina[a]

a) Natural Products & Food Research and Analysis – Pharmaceutical Technology (NatuRAPT), Department of Pharmaceutical Sciences, University of Antwerp, Universiteitsplein 1, 2610 Wilrijk, Antwerp, Belgium
b) Center for Medical Genetics, Faculty of Pharmaceutical, Biomedical, and Veterinary Sciences, University of Antwerp, Prins Boudewijnlaan 43 bus 6, 2650 Edegem, Antwerp, Belgium
c) Department of Cardiology, Antwerp University Hospital, Drie Eikenstraat 655, 2650 Edegem, Antwerp, Belgium
d) Laboratory of Physiopharmacology, Department of Pharmaceutical Sciences, University of Antwerp, Universiteitsplein 1, 2610 Wilrijk, Antwerp, Belgium

S2 Supplementary Material

Contents

Supplementary Material S2 1
- S2.1 HPLC-ECD Method for Quantification of Oleuropein and Hydroxytyrosol in Olive Extracts 2
- S2.2 Assessment of Dietary Intake 3
  - Questioned food items 3
  - Combination of food groups 3
  - Food categories of interest 4
  - Score calculation 4
- S2.3 Exploratory Outcomes 5
- S2.4 Side Effects 7
- References 9

S2.1 HPLC-ECD Method for Quantification of Oleuropein and Hydroxytyrosol in Olive Extracts

As quality control, the concentration of oleuropein (OLE) and hydroxytyrosol (HT) were determined in the study product with an *in-house* validated HPLC-ECD method adapted from Bayram et al. [1]. This method had a between-day precision of 4.03% (OLE) and 4.93% (HT), a within-day precision of 1.97% (OLE) and 4.41% (HT), and an accuracy of 99.78% (OLE) and 106.73% (HT). 100 mg of powder was suspended in 10 mL extraction solvent (1:1 EtOH:H_2O), placed in an ultrasonic bath for 30 min and centrifuged for 10 min at 2000*g. The supernatant was collected in a 100 mL volumetric flask. These extraction steps were performed two more times and the pooled supernatant was brought up to 100 mL with extraction solvent and diluted 25x for HPLC analysis in the extraction solvent. Reference solutions were prepared by dissolving 5 mg OLE and HT, obtained from Carl Roth (Karlsruhe, Germany) in 5 mL extraction solvent and serial diluted to 6 standard solutions ranging from 0.625 to 20 µg/mL. HPLC-ECD analysis was carried out with an Agilent 1620 Infinity HPLC from Agilent Technologies (Santa Clara, CA, USA) coupled to a Dionex Ultimate 3000 RS electrochemical detector using 6011 RS Ultra Coulometric Analytical cells with porous graphite working electrodes and solid-state palladium reference electrodes, both from Thermo Fisher Scientific (Waltham, MA, USA). An Eclipse Plus C18 column (4.6 x 100 mm, 3.5 µm) from Agilent (Santa Clara, CA, USA) was used as the stationary phase. Three mobile phases were prepared; (A) H_2O, (B) Acetonitrile, (C) MeOH, all of them containing 30 mM $LiClO_4$ (TCI, Zwijndrecht, Belgium). The pH of mobile phase A was adjusted to 3.1 with 70% $HClO_4$ (Merck, Darmstadt, Germany). The gradient is described as %A/%B/%C: 0 min 96/3/1, 4 min 85/10/5, 6 min 82.5/11.5/6, 11 min 72/18.5/9.5, 14 min 72/18.5/9.5, 17 min 0/85/15, 20 min 0/85/15, 23 min 96/3/1, 28 min 96/3/1. During the analysis, samples were kept at 4°C, 5 µL sample was loaded onto the column, and the column temperature was 40°C. The flow of 1.25 mL/min was applied and cell potential was set to 400 mV. Samples were analysed in triplicate.

S2.2 Assessment of Dietary Intake

Questioned food items

1. Water
2. Coffee, tea
3. Fruit juice, vegetable juice
4. Diet soft drinks
5. Soft drinks
6. Sports drinks
7. Energy drinks
8. Wine, champagne
9. Beer
10. Liquor
11. Other alcoholic beverages
12. Soy drinks, soy desserts
13. Milk in coffee, tea
14. Milk
15. Yoghurt, quark
16. Sweets, chocolate, candy bars
17. Biscuits, cookies
18. Patisserie
19. Seeds, seed spreads
20. Nuts, nut butters
21. Dried, candied fruit
22. Fruit
23. Breakfast cereals
24. White bread products
25. Brown, whole grain bread products
26. Sweet condiments
27. Cheese
28. Fish products
29. Cold cuts, processed meats
30. Eggs
31. Vegetarian products
32. Seafood
33. Fish
34. Organ meats
35. Rabbit and game
36. Poultry
37. Meat
38. Rice
39. Pasta
40. Diep-fried potato products
41. Backed or fried potatoes
42. Boiled and steamed potatoes
43. Legumes
44. Raw vegetables
45. Prepared vegetables
46. Chips, crisps, and deep-fried snacks
47. Mayonnaise, sauces based on it
48. Ketchup
49. Margarine, minarine
50. Butter, lard, smalt

Combination of food groups

- **Soft and sports drinks** = Soft drinks (5) + Sports drinks (6)
- **Alcohol** = Wine, champagne (8) + Beer (9) + Liquor (10) + other alcoholic beverages (11)
- **Candy, biscuits, chocolate, and patisserie** = Sweets, chocolate, candy bars (16) + Biscuits (17) + Patisserie (18)
- **Dough products** = White bread products (24) + Brown, whole grain bread products (25) + Rice (38) + pasta (39)
- **Vegetables** = Raw vegetables (44) + Prepared vegetables (45)
- **Potatoes**
  - **Potatoes (deep-fried + baked)** = Deep-fried potatoes (40) + Baked and fried potatoes (41)
  - **Potatoes (boiled)** = Boiled and steamed potatoes (42)

Food categories of interest

- **Polyphenol rich foods** = Coffee, tea (2) + Fruit juice, vegetable juice (3) + Seeds (20) + Nuts (20) + Dried and candied fruit (21) + Fruit (22) + Legumes (43) + Vegetables (combined group)

- **Foods rich in saturated and/or trans fatty acids** = Candy, biscuits, chocolate, and patisserie (combined group) + Cheese (27) + Cold cuts, processed meats (29) + Eggs (30) + Meat (37) + Potatoes (deep-fried + baked) (combined group) + Chips, crisps, and deep-fried snacks (46) + Mayonnaise (47) + Margarine, minarine (49) + Butter, lard, smalt (50)

- **Foods rich in sugar** = Fruit juices, vegetable juices (3) + Soft and sports drinks (combined group) + Energy drinks (7) + Soy drinks, soy desserts (12) + Milk (14) + Yoghurt (15) + Candy, biscuits, chocolate, and patisserie (combined group) + Dried and candied fruit (21) + Fruit (22) + Breakfast cereals (23) + Sweet condiments (26) + Ketchup (48)

Score calculation

The score of combined food groups or categories of interest is calculated by averaging the values of the individual food groups. A food group was excluded from the calculation of the mean if the value was missing or the score was equal to 0. This was done to avoid underestimation of the actual intake of the combined food group.

S2.3 Exploratory Outcomes

At baseline, no statistically significant differences were found between the intervention and placebo groups for the exploratory outcomes (Table S2.3.1).

Table S2.3.2 displays the mean value and standard error of insulin, C-peptide, CRP-US, creatinine, haemoglobin, waist circumference, and BMI at baseline and after eight weeks of treatment, together with the mean differences between the two time points and the p-value of the interaction term from the mixed model.

In the current trial, a reduction in insulin and C-peptide was observed in the intervention group, while the measurements increased in the control group. However, this change was non-significant.
Literature suggests that polyphenols could play a role in blood glucose management through the improvement of insulin resistance, increasing glucose uptake and the protection of β-cells in the liver [2–4]. Research on the effect of olive polyphenols on serum insulin levels as such is limited. A meta-analysis investigating the effect of extra virgin olive oil on glycaemic control reported a non-significant decrease in insulin levels. In this case, it is important to mention that the used trials differ in duration and study population. Making a comparison is, therefore, challenging [3].
CRP-US values in this trial were used to assess the inflammation status of the study subject at the start of the trial. Comparing these measurements between the two groups during the study did not result in a significant change. The results from literature on this topic are inconclusive as some trials report a significant decrease in CRP-US after the consumption of high polyphenol olive oil for three weeks [5], while others report no change in CRP-US levels [6,7].
As mentioned, creatinine and haemoglobin were measured to better interpret the HbA1c values. An exploratory investigation on these parameters during the trial gave a non-significant result. The meta-analysis by Razmpoosh et al. also reported no significant changes in creatinine levels in clinical trials investigating the effect of olive leaf extract on cardiovascular risk factors [7].
BMI and waist circumference were reported as descriptive parameters of the study population. They were also included in the statistical analysis, but no significant changes could be detected. However, an animal study reported that the administration of olive leaf extract to inactive mice on a high-fat diet suppressed the increases in fat mass and body weight [8].

In this trial, no significant changes in these exploratory parameters could be observed. However, it is important to note that the current clinical trial was not designed to investigate these parameters to their full extent. More search is therefore necessary.

Table S2.3.1 Baseline characteristics of study participants of the exploratory outcomes. Values are depicted as the mean with the corresponding standard deviation between brackets.

Characteristics	*Intervention (n = 28)*	*Control (n = 28)*	*p-value*
Insulin (pmol/L)	93.9 (60.4)	82.5 (42.9)	0.658
C-peptide (nmol/L)	0.9 (0.3)	0.9 (0.4)	0.973
CRP-US (mg/L)	1.7 (1.2)	2.7 (3.0)	0.481
Creatinine (mg/dL)	0.9 (0.1)	0.9 (0.1)	0.890
Haemoglobin (g/dL)	14.7 (1.0)	15.0 (1.3)	0.313

Table S2.3.2 Mean values with standard error of exploratory outcomes at baseline and after 8 weeks of treatment for the intervention and control group, together with the mean values with standard error and percentage of the raw difference in study outcomes for the intervention and control group, with the corresponding p-value of the mixed model interaction term.

	Intervention (n = 28)		*Control (n = 28)*		*Difference Intervention*		*Difference Control*		*p-value*
	Baseline	8 weeks	Baseline	8 weeks	Raw difference (SE)	%	Raw difference (SE)	%	
Insulin (pmol/L)	89.8 (11.1)	82.7 (13.2)	84.0 (8.3)	96.3 (13.5)	-7.1 (11.8)	-7.9%	12.3 (10.8)	14.6%	0.233
C-peptide (nmol/L)	0.86 (0.06)	0.85 (0.08)	0.91 (0.07)	0.98 (0.09)	-0.01 (0.07)	-1.16%	0.08 (0.06)	8.8%	0.376
CRP-US (mg/L)	1.7 (0.2)	1.8 (0.4)	2.8 (0.6)	2.6 (0.6)	0.1 (0.5)	7.6%	-0.2 (0.5)	-7.2%	0.643
Creatinine (mg/dL)	0.87 (0.03)	0.87 (0.03)	0.86 (0.03)	0.83 (0.02)	-0.01 (0.02)	-1.2%	-0.03 (0.01)	-3.5%	0.940
Haemoglobin (g/dL)	14.7 (0.2)	14.6 (0.2)	15.0 (0.2)	15.0 (0.3)	-0.1 (0.1)	-0.7%	0.01 (0.11)	0.1%	0.445
Waist circumference (cm)	100.3 (2.5)	100.1 (2.4)	103.8 (2.4)	103.0 (2.3)	-0.3 (0.7)	-0.3%	-0.8 (0.7)	-0.7%	0.565
BMI (kg/m²)	27.8 (0.9)	27.8 (0.9)	28.9 (1.0)	28.8 (1.0)	0.0 (0.1)	0.0%	-0.1 (0.1)	-0.3%	0.591

S2.4 Side Effects

No significant differences were observed in the prevalence of side effects between the two groups. 16/27 participants in the treatment group and 21/27 participants in the control group did not report any effects.

In the treatment group, joint pain (n = 1, light), sleeplessness (n = 3, light), hair loss (n = 1, light), obstipation (n = 1, light), acid reflux (n = 2, light), and dandruff (n = 1, light) were reported. In the control group, muscle soreness (n = 2, light and moderate) and globus (n = 1, light) were experienced. In both groups, participants complained of muscle pain (treatment n = 2, light and very severe; control n = 2, light and moderate), headache (treatment n = 2, light; control n = 2, light and moderate), and bloating (treatment n = 1, light; control n = 1, light).

If a side-effect was reported, most participants complained of one side-effect. Four participants who received the treatment intervention reported multiple side effects (obstipation and acid reflux, headache and acid reflux, muscle pain and bloating, and headache and dandruff), all indicated the severity level to be light. In the control group, one participant reported two side effects (moderate muscle pain and moderate muscle soreness) and another participant reported three side effects (muscle pain, muscle soreness and bloating), all indicated the severity level to be light.

The estimated glomerular filtration rate (eGFR) at the start of the study was 85.30 ± 6.31 mL/min/1.73 m^2 in the treatment group and 84.21 ± 7.82 mL/min/1.73 m^2 in the control group. These values were not significantly different (p-value = 0.667). Table S2.4 displays the mean value and standard error of the eGFR values at baseline and after eight weeks of treatment, together with the mean difference and the p-value of the interaction term from the mixed model.

Table S2.4: *Mean values with standard error of eGFR at baseline and after 8 weeks of treatment for the intervention and control group, together with the mean values with standard error and percentage of the difference in eGFR for the intervention and control group with the corresponding p-value of the mixed model interaction term.*

	Intervention		*Control*		*Difference Intervention*		*Difference Control*		*p-value*
	Baseline	8 weeks	Baseline	8 weeks	Raw difference (SE)	%	Raw difference (SE)	%	
eGFR (mL/min/1.73 m²)	85.12 (1.25)	84.89 (1.57)	84.26 (1.53)	85.41 (1.24)	0.42 (1.16)	0.49%	1.15 (0.85)	1.36%	0.539

References

[1] Bayram B, Ozcelik B, Schultheiss G, Frank J, Rimbach G. A validated method for the determination of selected phenolics in olive oil using high-performance liquid chromatography with coulometric electrochemical detection and a fused-core column. Food Chem 2013;138:1663–9. https://doi.org/10.1016/J.FOODCHEM.2012.11.122.

[2] de Bock M, Derraik JGB, Brennan CM, Biggs JB, Morgan PE, Hodgkinson SC, et al. Olive (Olea europaea L.) Leaf Polyphenols Improve Insulin Sensitivity in Middle-Aged Overweight Men: A Randomized, Placebo-Controlled, Crossover Trial. PLoS One 2013;8. https://doi.org/10.1371/journal.pone.0057622.

[3] Dehghani F, Morvaridzadeh M, Pizarro AB, Rouzitalab T, Khorshidi M, Izadi A, et al. Effect of extra virgin olive oil consumption on glycemic control: A systematic review and meta-analysis. Nutrition, Metabolism and Cardiovascular Diseases 2021;31:1953–61. https://doi.org/10.1016/J.NUMECD.2021.02.017.

[4] Kannan K, George JA, Sahadevan R, Kothari M, Sadhukhan S. Insights into one drug, multi-target aspects of polyphenols for diabetes management: in vitro, in vivo, and clinical evidence. Phytochemistry Reviews 2024:1–49. https://doi.org/10.1007/S11101-024-10047-9/FIGURES/3.

[5] Sarapis K, George ES, Marx W, Mayr HL, Willcox J, Esmaili T, et al. Extra virgin olive oil high in polyphenols improves antioxidant status in adults: a double-blind, randomized, controlled, cross-over study (OLIVAUS). Eur J Nutr 2022;61:1073–86. https://doi.org/10.1007/S00394-021-02712-Y/FIGURES/4.

[6] Correia M, Gomes ATPC, Moreira I, El Maghariki J, Mendes K, Correia MJ, et al. Unraveling the Extra Virgin Olive Oil Effect on Inflammation and on Gut and Saliva Microbiota. Biomolecules 2025;15:338. https://doi.org/10.3390/BIOM15030338.

[7] Razmpoosh E, Abdollahi S, Mousavirad M, Clark CCT, Soltani S. The effects of olive leaf extract on cardiovascular risk factors in the general adult population: a systematic review and meta-analysis of randomized controlled trials. Diabetol Metab Syndr 2022;14:151. https://doi.org/10.1186/S13098-022-00920-Y.

[8] Mikami T, Kim J, Park J, Lee H, Yaicharoen P, Suidasari S, et al. Olive leaf extract prevents obesity, cognitive decline, and depression and improves exercise capacity in mice. Sci Rep 2021;11:1–14. https://doi.org/10.1038/S41598-021-90589-6;SUBJMETA.
