# Supplementary material for: Evaluation of the effect of olive extracts on blood pressure and cardiovascular health markers in adults: Findings from a double-blind, placebo-controlled, randomised trial
Source: PLoS One. 2026 Mar 10;21(3):e0344278. doi: 10.1371/journal.pone.0344278 (PMC12974854; doi:10.1371/journal.pone.0344278)
Supplement: S3 File — (PDF) [file pone.0344278.s003.pdf]

Olive Trial

“Olive Polyphenols in Cardiovascular Prevention":
Effectiveness and Tolerance of a Commercially Available
Standardised Olive Extract (Tensiofytol®)
Compared to Placebo in Individuals With Elevated Blood Pressure;
A Randomised, Placebo-controlled, Double-blind Study

Study Protocol

The original study protocol was written in Dutch. This document was translated to English for publication using Microsoft Copilot (GPT-5) on November 4th 2025.

Microsoft. Copilot (GPT-5) [Large language model]. Microsoft; 2025.
Available from: https://copilot.microsoft.com

Contents

General Information 5
Sponsor 5
Principal Investigator – Medical Monitor 5
Researchers 5
Study Product 5
1. Protocol Summary 6
1.1 Protocol Synopsis 6
1.2 Trial Schema 7
1.3 Schedule of Activities 7
2. Introduction 8
2.1 Rationale 8
2.2 Summary of Benefits and Risks 9
3. Trial Objectives and Endpoints 10
3.1 Trial Objectives 10
3.2 Endpoints 10
4. Trial Design 11
4.1 Description of Trial Design 11
4.2 Rationale for Trial Design 12
4.2.1 Rationale for placebo 12
4.3 Access to Trial Intervention After End of Trial 13
4.4 Start and End of Trial 13
5. Trial Population 14
5.1 Selection of Trial Population 14
5.2 Rationale for Trial Population 14
5.3 Inclusion Criteria 14
5.4 Exclusion Criteria 14
5.5 Lifestyle Considerations 15
5.5.1 Meals and Dietary Restrictions 15
5.5.2 Caffeine, Alcohol, Tobacco, and Other Habits 15
5.5.3 Physical Activity 15
5.6 Screen Failures 15
5.7 Recruitment Methods 15
5.8 Compensation 15
6. Trial Intervention and Concomitant Therapy 16
6.1 Description of Trial Intervention 16

6.2 Rationale for Trial Intervention ... 16
6.3 Dosing and Administration ... 16
6.4 Treatment of Overdose ... 17
6.5 Preparation, Handling, Storage and Accountability ... 17
6.5.1 Preparation of Trial Intervention ... 17
6.5.2 Handling and Storage of Trial Intervention ... 17
6.5.3 Accountability of Trial Intervention ... 17
6.6 Participant Assignment, Randomisation and Blinding ... 17
6.6.1 Participant Assignment ... 17
6.6.2 Randomisation ... 17
6.6.3 Blinding and Unblinding ... 18
6.7 Trial Intervention Compliance ... 18
6.8 Concomitant Therapy ... 18
7. Discontinuation of Trial Intervention and Participant Withdrawal from Trial ... 19
7.1 Discontinuation of Trial Intervention ... 19
7.1.1 Criteria for Permanent Discontinuation of Trial Intervention ... 19
7.1.2 Temporary Discontinuation or Interruption of Trial Intervention ... 19
7.2 Participant Withdrawal for the Trial ... 19
7.3 Lost to Follow-Up ... 19
8. Trial Assessment and Procedures ... 20
8.1 Visits and Sampling ... 20
8.1.1 Screening visit ... 20
8.1.2 Intermediate Blood Pressure Measurement ... 20
8.1.3 End Visit ... 20
8.2 Sample Processing and Analyses ... 21
8.2.1 Sample Processing ... 21
8.2.2. Analyses ... 21
8.3 Data Management ... 22
8.4 Screening Procedures ... 22
8.5 Adverse Events and Serious Adverse Events ... 23
8.5.1 Definitions of AE and SAE ... 23
8.5.2 Time Period and Frequency for Collecting AE and SAE Information ... 23
8.5.3 Identifying and Recording of AEs and SAEs ... 23
8.5.4 Follow-up of AEs and SAEs ... 24
8.5.5 Reporting of SAEs ... 24
8.6 Pregnancy and Postpartum Information ... 24

8.6.1 Participants Who Become Pregnant During the Trial ... 24
8.6.2 Participants Whose Partners Become Pregnant ... 24
9. Statistical Considerations ... 25
9.1 Statistical Analysis ... 25
9.2 Sample Size Determination ... 25
10. General Considerations: Regulatory, Ethical, and Trial Oversight ... 26
10.1 Committees ... 26
10.2 Informed Consent Process ... 26
10.3 Data Protection ... 26
10.4 Funding ... 27
11. General Considerations: Risk Management and Quality Assurance ... 28
12. References ... 29

General Information

Sponsor

Antwerp University Hospital, Department of Cardiology
Drie Eikenstraat 655, 2650 Edegem (Belgium)
Prof. Dr. Johan Bosmans

University of Antwerp,
Natural Products & Food Research and Analysis – Pharmaceutical Technology (NatuRAPT)
Universiteitsplein 1, 2610 Wilrijk (Belgium)
Prof. Dr. Nina Hermans

Principal Investigator – Medical Monitor

Prof. Dr. Johan Bosmans
Antwerp University Hospital, Department of Cardiology
Drie Eikenstraat 655
2650 Edegem (België)

Researchers

**Name**	**Affiliation**
Prof. Dr. Nina Hermans	NatuRAPT<br>University of Antwerp
Dr. Tijs Bringmans	Department of Cardiology<br>Antwerp University Hospital
Dr. Ann Verhaegen	Department of Endocrinology, Diabetology and Metabolism<br>Antwerp University Hospital
Apr. Stef Lauwers	NatuRAPT<br>University of Antwerp

Study Product

Tensiofytol®

<u>Manufacturer</u>
N.V. Tilman®
Z.I. Sud 15, 5377 Baillonville (België)
+32(0)84 320 360 – info@tilman.be

1. Protocol Summary

1.1 Protocol Synopsis

Primary and Secondary Objectives and Endpoints

	Objective	Endpoint
**Primary**	Decrease in blood pressure	Systolic blood pressure*
**Secondary**	Decrease in blood pressure	Systolic blood pressure after 4 weeks*
	Decrease in blood pressure	Diastolic blood pressure*
	Change in markers of oxidative stress	oxLDL*, MDA*, GSH*
	Change in lipid profile	Total cholesterol*, HDL*, LDL*, non-HDL*, Remnant cholesterol*, Triglycerides*, Apolipoprotein A1*, Apolipoprotein B*, Lipoprotein(a)*
	Evaluation of side effects	Side effects
**Exploratory**	Change in parameters linked to metabolic syndrome.	Fasted glucose*, Hemoglobin A1c*, fasted insulin*, fasted C-peptide*
	Change in parameters linked to metabolic syndrome.	BMI* en waist circumference*
	Change in parameters linked to cardiovascular diseases	Hemoglobin*, Creatinine*, CRP-US*, Homocysteine*

* This always refers to the absolute difference compared to the baseline value after 8–9 weeks, unless stated otherwise

Study Design

Randomised, placebo-controlled, double-blind study investigating the effect, tolerance, and efficacy of Tensiofytol® compared to a placebo in individuals aged 18 years or older and younger than 78 years with elevated blood pressure.

Number of Arms

2 groups: Tensiofytol® and placebo

Blinding

Double-blind study; participants and researchers are unaware of the study group assignments.

Number of Participants

56 participants; 28 per group

1.2 Trial Schema

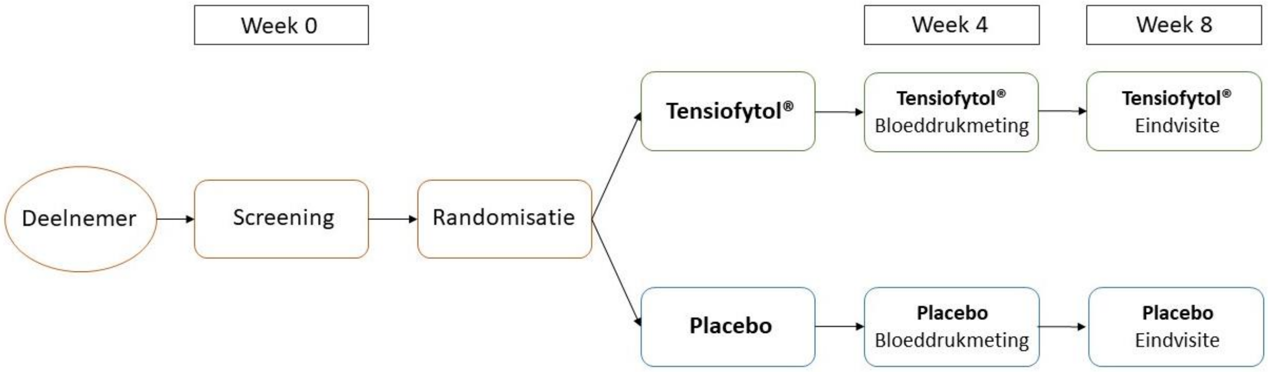

1.3 Schedule of Activities

Screening	Intermediate blood pressure measurement	End Visit
Week 0	Week 4 after start treatment	Week 8 – 9 after start treatment
• Review and signing of Informed Consent Form<br>• Complete contact form<br>• Blood pressure measurement<br>• Check inclusion and exclusion criteria<br>• Complete Food Frequency Questionnaire<br>• Complete start questionnaire<br>• Measure weight, height, and waist circumference<br>• Blood sampling	• Blood pressure measurement<br>• Adverse event inquiry	• Blood pressure measurement<br>• Complete Food Frequency Questionnaire<br>• Complete end questionnaire<br>• Measure weight and waist circumference<br>• Unblinding<br>• Provide book voucher and Tensiofytol® packaging if the participant received placebo during the study

2. Introduction

2.1 Rationale

Cardiovascular diseases (CVD), such as stroke or heart attack, are among the leading causes of death worldwide, with 17.9 million deaths per year [1]. High blood pressure is the main risk factor for developing CVD. It is also an important risk factor for other conditions such as chronic kidney disease and dementia [2].
In pathological situations, such as hypertension, large amounts of reactive oxygen species (ROS) are released. If endogenous antioxidants cannot neutralise these, it leads to oxidative stress. Oxidative stress is closely involved in the development and progression of CVD [3].
Studies indicate elevated levels of malondialdehyde (MDA), 8-iso-prostaglandin, and oxidised LDL (oxLDL, a key marker of oxidative lipid damage) [4–6].
Individuals with a combination of CVD risk factors — such as abdominal obesity, dyslipidemia, high blood pressure, and glucose intolerance — are considered to have metabolic syndrome (MetS), which greatly increases their risk of developing CVD [7].
Preventing CVD is therefore extremely important. Lifestyle changes, such as dietary adjustments, physical activity, and smoking cessation, as well as medication, can help [8]. Due to the high prevalence of CVD, further research into antihypertensive and antioxidant therapies is needed.

Polyphenols in olive extract have antioxidant properties and may therefore be useful in combating oxidative stress [9]. Previous studies (including one by the NatuRAPT research group using a combination of olive extract and red yeast rice) have shown that olive extract can lower elevated blood pressure [10,11]. Additionally, olive polyphenols have a positive effect on cholesterol levels [12–14]. Several years ago, EFSA (European Food Safety Authority) approved a health claim ("protects LDL from oxidation") for olive oil preparations related to a daily intake of 5 mg hydroxytyrosol [15,16]. Other studies have demonstrated an anti-hyperglycemic effect in individuals taking olive leaf extract for 12 weeks [17].

Olive polyphenols may therefore have a beneficial effect on various CVD risk factors.
In the current study, the effect of a standardised olive extract (Tensiofytol® – Tilman) will be investigated compared to placebo. Tensiofytol® is available over the counter, and the recommended daily dose contains 334 mg olive leaf dry extract and 106 mg olive fruit dry extract (*Olea europaea* L.; equivalent to 100 mg oleuropein and 20 mg hydroxytyrosol), divided over 2 capsules. For technical reasons, the same amount of product will be distributed over 3 capsules in this study.

2.2 Summary of Benefits and Risks

Benefit Summary

The dietary supplement in this study may help lower blood pressure and maintain healthy blood pressure, which, according to current medical insights, benefits the heart and blood vessels. During the study, the participant will receive information about their cardiovascular health and blood pressure values. In addition, the participant supports scientific research aimed at improving the treatment of elevated blood pressure.

Risk Summary

It cannot be guaranteed that the participant will personally gain direct benefit from participation. For example, the participant may be assigned to the placebo group, or it may turn out that the dietary supplement does not cause any change.

During the screening and final visit, blood samples will be taken. Blood sampling may cause minor discomfort, such as a small bruise. The blood draw will always be performed by a trained professional. The participant may experience side effects from the dietary supplement. There may also be risks and inconveniences that are currently unknown. During the study, participants will be asked about possible side effects. The participant may stop the study at any time, for example, if they experience discomfort from the supplement.

3. Trial Objectives and Endpoints

3.1 Trial Objectives

This study primarily aims to determine whether administering a commercially available standardised olive extract (Tensiofytol®) to individuals with elevated blood pressure can lead to a significant short-term reduction in blood pressure.

In addition, the study will examine whether the extract can cause changes in markers of oxidative stress and the lipid profile. The tolerance of the extract will also be evaluated.

Secondarily, the effect on other parameters related to metabolic syndrome and cardiovascular health will be assessed.

3.2 Endpoints

	**Objective**	**Endpoint**
**Primary**	Decrease in blood pressure	Systolic blood pressure*
**Secondary**	Decrease in blood pressure	Systolic blood pressure after 4 weeks*
	Decrease in blood pressure	Diastolic blood pressure*
	Change in markers of oxidative stress	oxLDL*, MDA*, GSH*
	Change in lipid profile	Total cholesterol*, HDL*, LDL*, non-HDL*, Remnant cholesterol*, Triglycerides*, Apolipoprotein A1*, Apolipoprotein B*, Lipoprotein(a)*
	Evaluation of side effects	Side effects
**Exploratory**	Change in parameters linked to metabolic syndrome.	Fasted glucose*, Hemoglobin A1c*, fasted insulin*, fasted C-peptide*
	Change in parameters linked to metabolic syndrome.	BMI* en waist circumference*
	Change in parameters linked to cardiovascular diseases	Hemoglobin*, Creatinine*, CRP-US*, Homocysteine*

* This always refers to the absolute difference compared to the baseline value after 8–9 weeks, unless stated otherwise.

4. Trial Design

4.1 Description of Trial Design

This is a randomised, placebo-controlled, double-blind study. It includes 56 participants, equally divided into 2 groups (28 participants per group). One group will receive Tensiofytol®, and the other will receive a placebo.
Participation in this study will take a maximum of 10 weeks. The potential participant will be invited for a screening visit. If eligible, the participant will be randomised. The study product will then be delivered to the participant as soon as possible. Intake of the study product must start no later than 1 week after the screening visit. The participant must then take the study product or placebo daily. An interim blood pressure measurement will take place 4 weeks after starting intake. The final visit will be scheduled 8 to a maximum of 9 weeks after starting intake. After the final visit, the participant will be unblinded, marking the end of participation.

Participants will be stratified by sex and then randomised into 2 groups. This will occur 1 to 2 days after the screening visit, once all results are available.
This study is double-blind. The investigators do not know which group the participant is assigned to. The participant will be informed of the treatment they received during the final visit, after all data have been collected. The randomisation list and unblinding at the final visit will be handled by a separate study staff member responsible solely for these tasks.

This is a single-centre study. Participants will be enrolled by researchers from the NatuRAPT research group.

Statistical analysis will be performed after all data from all participants have been collected. No interim analysis is planned.

4.2 Rationale for Trial Design

Randomised double-blind placebo-controlled studies are considered the gold standard.
The study is randomised so that unknown or unmeasurable variables do not lead to biased or incorrect results.
The study is double-blind to prevent researchers from treating the groups differently. This approach avoids bias in confirming the hypothesis and interpreting results. It also prevents a disproportionately large placebo effect.
The study is placebo-controlled so that psychosomatic effects do not influence the study outcome.

Studies investigating the effect of olive extract or combination preparations containing olive extract on blood pressure and cholesterol have observed changes in these parameters after an 8-week intervention [10,11]. The intervention in this study lasts 8 weeks but may be extended to 9 weeks for practical reasons.

The primary objective of this study is to assess the influence of the preparation on blood pressure. For this reason, both systolic and diastolic blood pressure will be measured during the screening visit, after 4 weeks, and at the final visit.
To observe changes in oxidative stress markers, MDA, oxLDL, and GSH will be measured in plasma samples collected during the screening and final visits.
At the final visit, participants will be asked about any experienced side effects to evaluate product tolerance.
Total cholesterol, HDL, LDL, non-HDL, remnant cholesterol, triglycerides, apolipoprotein A1, apolipoprotein B, and lipoprotein(a) will describe the participant's lipid profile. These parameters will be determined at the start and end of the study.
Additionally, BMI, waist circumference, fasting glucose, haemoglobin A1c, insulin, and fasting C-peptide will be monitored because abdominal obesity and glucose intolerance are components of MetS.
Haemoglobin, creatinine, ultra-sensitive C-reactive protein, and homocysteine will also be monitored as these parameters are linked to cardiovascular risk.

4.2.1 Rationale for placebo

The chosen placebo will have the same appearance and form as the study product. This way, participants will not know whether they are taking the placebo or the olive extract.

4.3 Access to Trial Intervention After End of Trial

At the final visit, the participant must return any remaining study product to the researcher. The study product, Tensiofytol®, is available over the counter. Participants may purchase the product themselves if they wish. If, after unblinding, the participant is found to have taken the placebo, they will receive one package of 112 capsules (for 8 weeks) free of charge.

4.4 Start and End of Trial

Study Preparation:	January – April 2021
Start of Recruitment:	June 2021
End of Recruitment:	When all participants have been enrolled (February 2024)
Analysis and Reporting:	2 months after the final visit of the last participant (April – May 2024)

5. Trial Population

5.1 Selection of Trial Population

The study investigates the effect of Tensiofytol® in individuals with elevated systolic blood pressure (≥130 mmHg).
Participants are aged ≥18 years and ≤77 years.

5.2 Rationale for Trial Population

Because the reduction in systolic blood pressure is the primary endpoint of this study, individuals with elevated systolic blood pressure were chosen as the study population. The product is currently commercially available as a supplement that helps maintain balanced blood pressure.
Participants are adults who are capable of providing informed consent for participation.
Individuals who do not meet these criteria may not be included in the study by exemption from these requirements.

5.3 Inclusion Criteria

To participate in this study, the potential participant must meet the following criterion:

1. An average systolic blood pressure ≥130 mmHg

5.4 Exclusion Criteria

Individuals meeting any of the following criteria will be excluded from participation in this study:

- Age <18 years or >78 years
- Smoking
- Triglyceride level >400 mg/dL
- More than 14 alcoholic drinks per week
- Chronic illness (e.g., diabetes, atherosclerosis, rheumatoid arthritis, …)
- Acute inflammation
- Pregnancy or intention to become pregnant during the study period
- Breastfeeding
- Regular use of dietary supplements (see §6.8 Concomitant Therapy)
- Use of (chronic) medication (see §6.8 Concomitant Therapy)

5.5 Lifestyle Considerations

5.5.1 Meals and Dietary Restrictions

There are no meal or diet restrictions.
At the start and end of the study, participants will complete a food questionnaire indicating the frequency with which they consume certain foods. This is to map the dietary habits of the study population.
Participants are asked not to change their eating habits during the study to avoid any influence on the results.

5.5.2 Caffeine, Alcohol, Tobacco, and Other Habits

Participants may not smoke or consume more than 14 alcoholic drinks per week.

5.5.3 Physical Activity

No restrictions regarding physical activity are imposed.
Participants are asked not to change the amount of physical activity during the study to avoid any influence on the results.

5.6 Screen Failures

If a potential participant is not eligible for this study, they will not be randomised. The participant will be informed by phone or email.
The option to schedule a new screening visit is not included in the protocol.

5.7 Recruitment Methods

Eligible patients from collaborating physicians/group practices will be briefly introduced to the study by the physician involved. If interested and after explicit consent, the contact details of these patients will be passed on to the NatuRAPT researchers. After providing detailed information, they will handle the practical details regarding possible inclusion.
The study will also be presented to UAntwerp staff via the website/email/Pintra. A flyer may provide an initial brief explanation. In case of slow recruitment, outreach may be expanded to UZA staff, pharmacies, and other cardiologists/general practitioners. Press releases will also be distributed. Doctors and pharmacists may receive compensation for referring eligible participants. For each included participant, the doctor or pharmacist will receive €20, or €150 for every 5 included participants.

5.8 Compensation

Participants will receive a €20 book voucher upon completion of the study, meaning at the end of the final visit. If, after unblinding, the participant is found to have taken the placebo, they will receive one package of 112 capsules (for 8 weeks) free of charge.

6. Trial Intervention and Concomitant Therapy

6.1 Description of Trial Intervention

The study product is the standardised olive extract Tensiofytol® from the manufacturer Tilman. It contains a recommended daily amount of 334 mg olive leaf dry extract and 106 mg olive fruit dry extract (*Olea europaea* L.; equivalent to 100 mg oleuropein and 20 mg hydroxytyrosol), divided over 3 capsules.
During the study, 3 capsules must be taken orally each day with a glass of water at dinner for 8 weeks or until the evening before the final visit (maximum 9 weeks after starting intake). The participant will receive a container with 210 capsules, sufficient for 10 weeks of treatment. The packaging must be stored at room temperature (15–25 °C).
The packaging is labelled with the following label:

Olijfstudie UAntwerpen 2021:
Olijfpolyfenolen in cardiovasculaire preventie
olijfstudie@uantwerpen.be
Orale inname. 3 capsules/dag bij het avondmaal
Deelnemer xxx
Enkel voor gebruik tijdens de klinische studie.
Bewaren op kamertemperatuur (15-25 °C), op een droge plaats buiten direct zonlicht.
Buiten het bereik van kinderen bewaren, in de originele verpakking.
Inhoud: 210 capsules.
Vervaldatum: dd /maand / jjjj

The placebo contains magnesium stearate, calcium phosphate, colloidal silica, microcrystalline cellulose, and talc.
During the study, 3 capsules must be taken orally each day with a glass of water at dinner, for 8 weeks or until the evening before the final visit (maximum 9 weeks after starting intake).
The participant will receive a container with 210 capsules, sufficient for 10 weeks of treatment.
The packaging must be stored at room temperature (15–25 °C).
The label and appearance are identical to those of the study product.

6.2 Rationale for Trial Intervention

The dose and administration correspond to the posology of the commercially available product Tensiofytol®.
For practical reasons, the same amount of product is divided over 3 capsules instead of 2 capsules as available in the public pharmacy.

6.3 Dosing and Administration

The participant must take 3 capsules orally at the same time. This should be done with the evening meal for 8 weeks or until the evening before the final visit (maximum 9 weeks after starting intake).
The study product should be taken while sitting upright and with a glass of water.
No dose adjustments are allowed.

6.4 Treatment of Overdose

In case of overdose, contact must be made with the principal investigator – medical monitor. They will assess the situation. If they are not reachable, the participant will be referred to emergency services. The UZA emergency department can be contacted at:
+32(0)2 831 46 07

6.5 Preparation, Handling, Storage and Accountability

6.5.1 Preparation of Trial Intervention

The study product and placebo are prepared by the manufacturer. The containers are shipped by mail to the NatuRAPT researchers.
The content of oleuropein and hydroxytyrosol is verified by NatuRAPT (see protocol "Quantification of Olive Polyphenols in Extract").

6.5.2 Handling and Storage of Trial Intervention

The containers are shipped by mail and stored at room temperature (15–25 °C) by the NatuRAPT research group.
When distributed to participants, the product will be handed over physically or sent by mail.
The products remain stable for at least 3 years after preparation.
Stability is monitored by the manufacturer.

6.5.3 Accountability of Trial Intervention

The product is delivered to the participant by the NatuRAPT researchers. During the final visit, any remaining study product is returned to the researchers and stored by them.
Delivery and return of the study products are recorded in the "Drug Dispensation & Accountability Log."

6.6 Participant Assignment, Randomisation and Blinding

6.6.1 Participant Assignment

Participants will be stratified by sex and randomised after collecting and reviewing the inclusion criteria.

6.6.2 Randomisation

Participant numbers will be stratified into two groups by sex and then randomised using an online tool ("Clinical Trial Randomization Tool." The National Cancer Institute's Division of Cancer Prevention, https://ctrandomization.cancer.gov), resulting in two lists of numbers. The aim is to include an equal number of men and women in each group.
Participants will be assigned to these lists in the order of inclusion and thus randomised accordingly.

6.6.3 Blinding and Unblinding

The packaging will be numbered by a researcher. These numbers will then be used for randomisation and correspond to participant numbers.
During the final visit, the participant's treatment will be unblinded by this researcher. Each participant has a separate envelope indicating their treatment, allowing unblinding of an individual participant without revealing the treatment of others.
This researcher does not collect participant data or perform measurements on participant samples. Randomisation, blinding, and unblinding are the only tasks assigned to this researcher.
Unblinding will be documented in the unblinding register.

Emergency Unblinding

In exceptional circumstances, such as severe adverse events or hospitalisation, the participant's treatment may be unblinded.
The participant can contact a study staff member. The individual envelope for that participant can be opened to reveal the treatment.
Unblinding must always be reported to the principal investigator – medical monitor.

6.7 Trial Intervention Compliance

Compliance will be monitored in the "Drug Dispensation & Accountability Log."
At the end of the study, the remaining number of capsules will be counted and subtracted from the number originally provided. This will determine the number of capsules taken. The ratio of capsules actually taken to the expected number will be expressed as a percentage to reflect compliance.

6.8 Concomitant Therapy

Participants may not use dietary supplements and/or chronic medication.
Participation is possible if the intake of dietary supplements is stopped at least 10 days before the screening visit and throughout the study duration.
For chronic medication use, an individual assessment will determine whether the medication could affect the study endpoints or whether taking the study product is contraindicated.
For example, participation is possible for individuals using antihypertensive drugs if the dosage is stable and will not change during the study. The participant must still meet the inclusion criterion.
Individuals who have been using, for example, a PPI for several months may also participate.

7. Discontinuation of Trial Intervention and Participant Withdrawal from Trial

7.1 Discontinuation of Trial Intervention

The treatment may be discontinued if the participant or the investigator wishes.

7.1.1 Criteria for Permanent Discontinuation of Trial Intervention

The treatment will be stopped if the participant meets any exclusion criteria during the study, such as starting smoking or changing their diet.
The treatment may also be stopped if the participant wishes, experiences discomfort from the treatment, or for any other reason.
The participant must return any remaining study product capsules.

7.1.2 Temporary Discontinuation or Interruption of Trial Intervention

Temporary discontinuation of treatment is not allowed. Stopping the treatment will result in exclusion from the study.

7.2 Participant Withdrawal for the Trial

The participant may withdraw from the study at any time, for any reason, and without any consequences.
The participant must return any remaining study products.

7.3 Lost to Follow-Up

Due to the short treatment duration, the proportion of participants who do not complete the study is expected to be low. Additional participants will be recruited to ensure sufficient statistical validity. Participants who do not attend the final visit or fail to complete the study will not be replaced.

8. Trial Assessment and Procedures

8.1 Visits and Sampling

The study consists of 3 visits: a screening visit, an intermediate blood pressure measurement, and a final visit

8.1.1 Screening visit

Potential candidates are invited for a screening visit. The visit includes the following actions:

- Review the ICF, answer questions, and sign (see 10.2 Informed Consent)
- Complete contact form (see 8.4 Screening Procedures)
- Measure blood pressure 3 times (see protocol "Blood Pressure Measurement")
- Check inclusion and exclusion criteria using the inclusion checklist (see 8.4 Screening Procedures)
- Complete Food Frequency Questionnaire (FFQ)
- Complete baseline questionnaire (see 8.4 Screening Procedures)
- Measure weight, height, and waist circumference (see 8.4 Screening Procedures)
- Blood sampling by a nurse (34 mL) (see protocol "Blood Sampling")
    - 2x Serum tube (2 × 7 mL)
    - 3x EDTA tube (3 × 4 mL)
    - 1x Glucose tube (1 × 4 mL)
    - 1x Homocysteine tube (1 × 4 mL)

8.1.2 Intermediate Blood Pressure Measurement

Four weeks after starting intake, blood pressure will be measured (see protocol "Blood Pressure Measurement").
The results of the measurements will be recorded in the participant's file.

8.1.3 End Visit

Eight to a maximum of nine weeks after starting intake, the participant will be invited for a final visit. The visit includes the following actions:

- Measure blood pressure 3 times (see protocol "Blood Pressure Measurement")
- Complete Food Frequency Questionnaire (FFQ)
- Complete final questionnaire
- Measure weight and waist circumference
- Blood sampling by a nurse (34 mL) (see protocol "Blood Sampling")
    - 2x Serum tube (2 × 7 mL)
    - 3x EDTA tube (3 × 4 mL)
    - 1x Glucose tube (1 × 4 mL)
    - 1x Homocysteine tube (1 × 4 mL)
- Unblinding
- Provide book voucher
- Return Tensiofytol® packaging if the participant received placebo during the study

8.2 Sample Processing and Analyses

8.2.1 Sample Processing

Two EDTA tubes will be centrifuged and divided into aliquots of plasma, red blood cells, and buffy coats according to the protocol “Sample Collection.”
One serum tube will be centrifuged and divided into serum aliquots according to the same protocol.

8.2.2. Analyses

Glutathione (GSH)

GSH levels will be determined in red blood cells using HPLC-ECD according to the protocol “GSH Determination.”
The analysis will be performed by NatuRAPT.

MDA

MDA will be measured in plasma using ELISA kit E-EL-0060 from Elabscience® according to the provided protocol.
The analysis will be performed by NatuRAPT.

oxLDL

oxLDL will be measured in plasma using ELISA kit 10-1143-01 from Mercodia according to the provided protocol.
The analysis will be performed by NatuRAPT.

Blood Parameters

The remaining blood samples will be sent to:
Clinical Laboratory AML
Emiel Vloorsstraat 9
2020 Antwerp (Belgium)

The following analyses will be requested:

- Hemoglobin
- Creatinine and eGFR
- Total cholesterol, HDL, LDL, Cholesterol/HDL ratio, non-HDL, triglycerides, Apo A1, Apo B, Lp(a), CRP-US, and homocysteine
- Fasting glucose, Hemoglobin A1c, HbA1c (IFCC)
- Fasting insulin and fasting C-peptide

8.3 Data Management

Research data will be collected and managed using REDCap electronic data capture tools, hosted by the University of Antwerp.
REDCap (Research Electronic Data Capture) is a secure, web-based software platform designed to support data capture for research studies, providing:

1. An intuitive interface for validated data entry;
2. Audit trails for tracking data manipulation and export procedures;
3. Automated export procedures for data downloads to common statistical packages;
4. Procedures for data integration and interoperability with external sources.

8.4 Screening Procedures

During screening, the following specific actions will be performed:

1. Obtain informed consent (see §10.2 Informed Consent)

2. Complete contact form
   The following contact details will be collected from the potential participant:
   - First and last name
   - Address
   - Phone number and email address

3. Check inclusion and exclusion criteria
   - Blood pressure measurement (see protocol "Blood Pressure Measurement")
   - Complete inclusion checklist by the investigator
   - Complete baseline questionnaire by the participant

4. Collect physical parameters
   - Waist circumference
   - Height
   - Weight

8.5 Adverse Events and Serious Adverse Events

8.5.1 Definitions of AE and SAE

Adverse Event (AE)

Any undesirable or unfavorable medical occurrence, including abnormal signs (e.g., abnormal physical examination or laboratory findings), symptom, or illness in a participant, that is temporarily associated with the participant's involvement in the study, whether or not considered related to study participation.

Serious Adverse Event

Any event that:

- Results in death
- Is life-threatening
- Requires hospitalisation or prolongs hospitalisation
- Causes persistent or significant disability or incapacity
- Results in a congenital anomaly or birth defect
- Is considered by the investigators to pose significant hazards

8.5.2 Time Period and Frequency for Collecting AE and SAE Information

Throughout the study, from the start of intake until the final visit, AE and SAE data can be collected.
After the study ends, participants will not be actively asked whether AEs or SAEs occurred.
During the final visit, a questionnaire will be completed asking about side effects or discomfort.
At the intermediate blood pressure measurement, there is an in-person contact moment where participants are asked how they tolerate the study product.

8.5.3 Identifying and Recording of AEs and SAEs

Participants have the contact details of the investigators and the principal investigator – medical monitor. AEs and SAEs can be reported this way. In case of an SAE, investigators and the medical monitor must be notified immediately.

At in-person visits, participants are asked how they tolerate the study product. Any comments are noted.
During the final visit, participants complete the final questionnaire, which asks about side effects and their severity. If possible, a start and end date for each complaint will be specified.

AEs and SAEs are recorded in the "Adverse Events / Serious Adverse Events" register. For each event, a description, start and end date, outcome, severity, and actions taken are documented.
It is then assessed whether the AE meets the criteria for SAE and whether it may be related to the treatment.
This assessment is done in consultation with the principal investigator – medical monitor.

8.5.4 Follow-up of AEs and SAEs

After the study ends, participants will not be actively asked whether AEs or SAEs occurred.
If a complaint began during study participation but has not been resolved, no end date will be recorded.

8.5.5 Reporting of SAEs

If an SAE occurs, the Serious Adverse Event reporting form must be completed.
An SAE must be reported to the principal investigator – medical monitor as soon as possible.
The SAE will also be reported to the Ethics Committee.

8.6 Pregnancy and Postpartum Information

8.6.1 Participants Who Become Pregnant During the Trial

The participant must inform us of the pregnancy, which will result in exclusion from the study.
Since no adverse effects have been recorded, no follow-up will be provided.

8.6.2 Participants Whose Partners Become Pregnant

This does not pose any problem for participation.

9. Statistical Considerations

9.1 Statistical Analysis

Demographic data and baseline values will be used to describe the study groups.
The difference in each parameter compared to baseline will be analyzed using ANCOVA by treatment group, adjusted for baseline values. An α-level of 0.025 will be applied for the primary analysis. For secondary and exploratory analyses, an α-level of 0.05 will be used.

The absolute change from baseline in blood pressure over time will be studied using Linear Mixed Models. Baseline value, treatment group, time point, and the interaction between treatment group and time point will be fixed variables. Participant ID will be a random variable.

As sensitivity or exploratory analyses, predictors such as sex, age, baseline values, or compliance may be added to the models to assess whether they have a significant effect.

Subgroup analyses may also be performed, and other continuous parameters can be studied using the same method.

No interim analysis is planned.

9.2 Sample Size Determination

Previous studies observed a reduction in systolic blood pressure of 10.4 ± 11.4 mmHg after intake of olive extract [10,11]. A reduction in systolic blood pressure is the primary endpoint and is therefore used to determine the sample size.
Since another study will examine a different primary endpoint in participants from the placebo group of this study, an α-level of 0.025 is used. The planned power is 80%.
It is calculated that at least 25 participants per group are needed to detect this effect. This calculation was performed using G*Power 3.1. Considering an anticipated dropout rate of 10%, 28 participants per group (56 in total) will be recruited for this study.

10. General Considerations: Regulatory, Ethical, and Trial Oversight

10.1 Committees

The study is reviewed and reported to the Medical Ethics Committee of UZA/UAntwerp.

10.2 Informed Consent Process

Before the screening visit, the potential participant will receive the informed consent form (ICF) by mail. The participant must read this before the screening.
During the screening visit, the researcher will review the ICF with the participant, who may ask questions.
If the participant agrees, the ICF will be signed in duplicate. Both the researcher and the participant will receive a copy.

10.3 Data Protection

The identity and participation in this study will be treated with strict confidentiality. The participant will not be identified by name or in any other way in files, results, or publications related to the study. To guarantee privacy, data will be pseudonymized. All further processing will be carried out on these pseudonymized data. The link between the pseudonym and the individual will be retained only to allow certain information to be communicated back to the participant in their own interest.
The participant consents to the use of personal data collected for this study. This consent can be withdrawn at any time. If participation ends prematurely, the original consent allows the use of data collected during the period the participant was actively involved in the study. Only individuals directly involved in the study will have access to personal data. Data will not be shared with third parties. Researchers will retain the data for a period of 25 years.
The participant has the right to ask the researcher which data are collected and for what purpose. The participant may request corrections or deletion of certain data or ask that data no longer be used. All data will be handled in accordance with the "Directive on the protection of individuals regarding the processing of personal data" and applicable national legislation, including the European General Data Protection Regulation (GDPR – EU2016/679) and Belgian law implementing this regulation. Questions regarding data management can be addressed to the study physician or treating physician.
If the participant feels that their rights regarding personal data are not adequately respected, they may contact the Data Protection Officer, who will take appropriate measures. The participant also has the right to file a complaint with the Belgian Data Protection Authority. More information: https://www.uza.be/privacy.html.

10.4 Funding

This study was partly funded by the chair "Tilman – Olive Polyphenols and Cardiovascular Health." Although Tilman funds the chair (budget includes salary for a part-time researcher, participant recruitment, study execution, report writing, blood sampling, participant travel costs, and publication costs), this is a non-commercial study with UAntwerp as the sponsor.

There was no contractual commitment regarding the study and the results.
Tilman had no methodological or any other input in the design, execution or reporting of this trial. Tilman had no access to the detailed study protocol or raw study data.
Before starting the study, it was agreed that the results would be published, regardless of the outcome.

11. General Considerations: Risk Management and Quality Assurance

The quality of the study products is guaranteed by the manufacturer, Tilman.
The content of oleuropein and hydroxytyrosol in the study products will be verified by NatuRAPT.

12. References

[1] WHO. Fact-Sheet Cardiovascular diseases 2017 n.d. https://www.who.int/news-room/fact-sheets/detail/cardiovascular-diseases-(cvds) (accessed October 25, 2023).

[2] Fuchs FD, Whelton PK. High Blood Pressure and Cardiovascular Disease. Hypertension 2020;75:285–92. https://doi.org/10.1161/HYPERTENSIONAHA.119.14240.

[3] Dubois-deruy E, Peugnet V, Turkieh A, Pinet F. Oxidative stress in cardiovascular diseases. Antioxidants 2020;9:1–15. https://doi.org/10.3390/antiox9090864.

[4] Armutcu F, Ataymen M, Atmaca H, Gurel A. Oxidative stress markers, C-reactive protein and heat shock protein 70 levels in subjects with metabolic syndrome. Clin Chem Lab Med 2008;46:785–90. https://doi.org/10.1515/CCLM.2008.166.

[5] Fujita K, Nishizawa H, Funahashi T, Shimomura I, Shimabukuro M. Systemic Oxidative Stress is Associated With Visceral Fat Accumulation and the Metabolic Syndrom. Circulation Journal 2006;70:1437–42. https://doi.org/https://doi.org/10.1253/circj.70.1437.

[6] Chae JS, Kim OY, Paik JK, Kang R, Seo WJ, Jeong TS, et al. Association of Lp-PLA 2 activity and LDL size with interleukin-6, an inflammatory cytokine and oxidized LDL, a marker of oxidative stress, in women with metabolic syndrome. Atherosclerosis 2011;218:499–506. https://doi.org/10.1016/j.atherosclerosis.2011.06.036.

[7] Grundy SM, Brewer HB, Cleeman JI, Smith SC, Lenfant C. Definition of Metabolic Syndrome: Report of the National Heart, Lung, and Blood Institute/American Heart Association Conference on Scientific Issues Related to Definition. Circulation, vol. 109, 2004, p. 433–8. https://doi.org/10.1161/01.CIR.0000111245.75752.C6.

[8] Visseren F, Mach F, Smulders YM, Carballo D, Koskinas KC, Bäck M, et al. 2021 ESC Guidelines on cardiovascular disease prevention in clinical practice. Eur Heart J 2021;42:3227–337. https://doi.org/10.1093/eurheartj/ehab484.

[9] Hermans N, Van der Auwera A, Breynaert A, Verlaet A, De Bruyne T, Van Gaal L, et al. A red yeast rice-olive extract supplement reduces biomarkers of oxidative stress, OxLDL and Lp-PLA2, in subjects with metabolic syndrome: A randomised, double-blind, placebo-controlled trial. Trials 2017;18. https://doi.org/10.1186/s13063-017-2058-5.

[10] Verhoeven V, Van der Auwera A, Van Gaal L, Remmen R, Apers S, Stalpaert M, et al. Can red yeast rice and olive extract improve lipid profile and cardiovascular risk in metabolic syndrome?: A double blind, placebo controlled randomized trial. BMC Complement Altern Med 2015;15. https://doi.org/10.1186/s12906-015-0576-9.

[11] Hermans MP, Lempereur P, Salembier JP, Maes N, Albert A, Jansen O, et al. Supplementation effect of a combination of olive (Olea europea L.) leaf and fruit extracts in the clinical management of hypertension and metabolic syndrome. Antioxidants 2020;9:1–15. https://doi.org/10.3390/antiox9090872.

[12] Rigacci S, Stefani M. Nutraceutical properties of olive oil polyphenols. An itinerary from cultured cells through animal models to humans. Int J Mol Sci 2016;17. https://doi.org/10.3390/ijms17060843.

[13] Peyrol J, Riva C, Amiot MJ. Hydroxytyrosol in the prevention of the metabolic syndrome and related disorders. Nutrients 2017;9. https://doi.org/10.3390/nu9030306.

[14] Hernáez Á, Remaley AT, Farràs M, Fernández-Castillejo S, Subirana I, Schröder H, et al. Olive oil polyphenols decrease LDL concentrations and LDL atherogenicity in men in a randomized

controlled trial. Journal of Nutrition 2015;145:1692–7. https://doi.org/10.3945/jn.115.211557.

[15] EFSA Panel on Dietetic Products N and A (NDA). Scientific Opinion on the substantiation of health claims related to polyphenols in olive. EFSA Journal 2011;9:2033. https://doi.org/10.2903/j.efsa.2011.2033.

[16] Weinbrenner T, Fitó M, De La Torre R, Saez GT, Rijken P, Tormos C, et al. Human Nutrition and Metabolism Olive Oils High in Phenolic Compounds Modulate Oxidative/Antioxidative Status in Men 1. J Nutr 2004;134:2314–21. https://doi.org/https://doi.org/10.1093/jn/134.9.2314.

[17] de Bock M, Derraik JGB, Brennan CM, Biggs JB, Morgan PE, Hodgkinson SC, et al. Olive (Olea europaea L.) Leaf Polyphenols Improve Insulin Sensitivity in Middle-Aged Overweight Men: A Randomized, Placebo-Controlled, Crossover Trial. PLoS One 2013;8. https://doi.org/10.1371/journal.pone.0057622.
